# Sugarcane transgenics expressing MYB transcription factors show improved glucose release

**DOI:** 10.1186/s13068-016-0559-1

**Published:** 2016-07-15

**Authors:** Charleson R. Poovaiah, William P. Bewg, Wu Lan, John Ralph, Heather D. Coleman

**Affiliations:** Department of Biology, Syracuse University, Syracuse, NY 13244 USA; Center for Tropical Crops and Biocommodities, Queensland University of Technology, Brisbane, QLD 4000 Australia; US Department of Energy, Great Lakes Bioenergy Research Center (GLBRC), Wisconsin Energy Institute, University of Wisconsin, Madison, WI 53726 USA; Department of Biological System Engineering, University of Wisconsin, Madison, WI USA; Department of Biochemistry, University of Wisconsin, Madison, WI 53726 USA

**Keywords:** Sugarcane bagasse, Bioethanol, Biofuel, Lignin modification

## Abstract

**Background:**

Sugarcane, a tropical C4 perennial crop, is capable of producing 30–100 tons or more of biomass per hectare annually. The lignocellulosic residue remaining after sugar extraction is currently underutilized and can provide a significant source of biomass for the production of second-generation bioethanol.

**Results:**

*MYB31* and *MYB42* were cloned from maize and expressed in sugarcane with and without the UTR sequences. The cloned sequences were 98 and 99 % identical to the published nucleotide sequences. The inclusion of the UTR sequences did not affect any of the parameters tested. There was little difference in plant height and the number of internodes of the *MYB*-overexpressing sugarcane plants when compared with controls. *MYB* transgene expression determined by qPCR exhibited continued expression in young and maturing internodes. *MYB31* downregulated more genes within the lignin biosynthetic pathway than *MYB42*. *MYB31* and *MYB42* expression resulted in decreased lignin content in some lines. All MYB42 plants further analyzed showed significant increases in glucose release by enzymatic hydrolysis in 72 h, whereas only two MYB31 plants released more glucose than control plants. This correlated directly with a significant decrease in acid-insoluble lignin. Soluble sucrose content of the MYB42 transgenic plants did not vary compared to control plants.

**Conclusions:**

This study demonstrates the use of *MYB* transcription factors to improve the production of bioethanol from sugarcane bagasse remaining after sugar extraction.

**Electronic supplementary material:**

The online version of this article (doi:10.1186/s13068-016-0559-1) contains supplementary material, which is available to authorized users.

## Background

Sugarcane (*Saccharum* spp. Hybrids) is a C4 perennial crop grown in tropical and subtropical climates that is capable of producing large amounts of dry biomass per hectare annually [1]. It has a high capacity to convert light energy to biomass, which, along with efficient water and nitrogen use, makes it suitable to be grown in marginal land areas [2, 3]. Although best known for accumulating and storing high concentrations of sucrose in its stem, and its use for sugar production, sugarcane also produces abundant lignocellulosic residues that remain after sugar extraction. This material is currently underutilized, but holds the potential to act as a renewable source for biofuel production [4].

One of the main constraints in the production of biofuels is recalcitrance of feedstocks to enzymatic hydrolysis [5]. Lignin, a major component of the secondary cell wall, imparts mechanical support to the plant, enhances water transport, and protects the plants against pathogens [6], but at the same time acts as a barrier in the extraction of fermentable sugars from the lignocellulosic biomass due to its ability to resist degradation. Lignin is synthesized through the phenylpropanoid pathway by the oxidative polymerization of the monolignols—*p*-coumaryl, coniferyl and sinapyl alcohols. The resulting units of monolignol, when incorporated into the lignin polymer, are called *p*-hydroxyphenyl (H), guaiacyl (G) and syringyl (S) subunits, respectively [6, 7]. Pretreatment steps to remove lignin lead to the formation of compounds that inhibit saccharification and fermentation [5].

Lignin biosynthesis is regulated by the action of different transcription factors, including members of the R2-R3 MYB transcription factor family [8]. These transcription factors bind to the AC elements in the promoters of lignin biosynthetic genes and drive xylem-specific expression [9]. Among these transcription factors are *ZmMYB31* and *ZmMYB42*, both of which are involved in the regulation of lignin biosynthesis in *Zea mays*. Expression of *ZmMYB31* and *ZmMYB42* independently in Arabidopsis resulted in reduced expression of lignin biosynthetic genes and led to a significant decrease in lignin content [10–12]. This resulted in greater release of fermentable sugars during enzymatic hydrolysis from the transgenics when compared with controls [10, 11, 13]. Recent research found that the overexpression of *PvMYB4* in switchgrass reduced lignin content through the downregulation of lignin biosynthetic genes, which in turn increased the saccharification of these plants threefold [14, 15]. Phylogenetic analysis of *PvMYB4* showed that it was more closely related to *ZmMYB31*, *ZmMYB42* and *ZmMYB38* than the other MYB transcription factors comprising subgroup G4 [11, 16].

In this report, *ZmMYB31* and *ZmMYB42* were overexpressed in sugarcane to emulate previous findings [10–12] and to improve saccharification, as this characteristic would benefit the production of second-generation bioethanol from, and increase the monetary value of, sugarcane bagasse.

## Results

### Alignment of cloned *ZmMYB31* and *ZmMYB42* sequences

*ZmMYB31* and *ZmMYB42* were cloned from maize and transformed into sugarcane under the control of a maize ubiquitin promoter. There was 99 % identity between the published *MYB31* sequence and the cloned sequence for both nucleotide and protein sequences, whereas the published *MYB42* sequence and the cloned sequences had 98 % nucleotide identity and 99 % protein identity (see Additional file 1 for nucleotide and amino acid sequence alignment). There was 100 % amino acid sequence identity of the R2 and R3 domains between the cloned maize *MYB31* and *MYB42* genes and published sequences [12]. There were few differences in amino acid sequence within the C-terminal end of the translated MYB31 and MYB42 proteins and also in the nucleotide sequences of the 5′ and 3′ UTR sequences between cloned and published *MYB31* sequences (see Additional file 1).

### Phenotype of MYB plants

Seven lines were generated for each of the four constructs. Additionally, *GFP* was cloned in place of *MYB* in the same vector and the plants generated used as transgenic controls. Plants were confirmed as harboring the transgene of interest before being transferred to the greenhouse. Following 9 months of growth, the plants were harvested for analysis. Phenotypic measurements, including plant height, number of internodes, internode diameter of the third internode, and average internode length were determined at the time of harvest (Table 1). A *z* score was calculated based on the average results of the GFP transgenic control plants. Any MYB plant with a *z* score greater than 2 or less than −2, indicating more than two standard deviations from the control group average, was considered to be different from controls. Overall, there was little difference in the height or number of internodes relative to controls and, in general, any changes seen in internode length were offset by changes in the number of internodes. MYB31ORF 7 was taller than controls and MYB42UTR 30 was shorter. MYB3ORF 2 was the only plant with a significant change in internode number, having fewer than the GFP control group (Table 1).

**Table 1:** Phenotypic measurements of MYB-expressing sugarcane

Plant	Height (cm)		Total number of internodes		Third internode diameter (mm)		Average internode length (cm)	
GFP	129 ± 24.26		15 ± 2.16		14.18 ± 0.36		8.56 ± 0.60	
		z score		z score		z score		z score
MYB31ORF								
13	114	−0.62	14	−0.46	12.00	−*5.99*	8.14	−0.7
11	132	0.12	13	−0.93	14.29	0.29	10.15	*2.63*
2	100	−1.20	10	−*2.31*	11.63	−*7.00*	10.00	*2.38*
7	205	*3.13*	15	0.00	12.93	−*3.44*	13.67	*8.45*
1	140	0.45	14	−0.46	8.76	−*14.87*	10.00	*2.38*
8	158	1.20	14	−0.46	12.45	−*4.75*	11.29	*4.51*
9	159	1.24	15	0.00	11.16	−*8.29*	10.60	*3.37*
MYB31UTR								
27	140	0.45	16	0.46	12.57	−*4.42*	8.75	0.31
2	128	−0.04	13	−0.93	11.76	−*6.65*	9.85	*2.12*
18	131	0.08	13	−0.93	14.45	0.73	10.08	*2.51*
11	160	1.28	15	0.00	12.45	−*4.75*	10.67	*3.48*
12	147	0.74	14	−0.46	11.80	−*6.54*	10.50	*3.21*
7	136	0.29	15	0.00	15.27	*2.98*	9.07	0.83
20	108	−0.87	15	0.00	12.79	−*3.82*	7.20	−*2.26*
MYB42ORF								
14	162	1.36	17	0.93	13.52	−1.82	9.53	1.6
16	130	0.04	17	0.93	17.28	*8.49*	7.65	−1.52
23	134	0.21	15	0.00	14.39	0.57	8.93	0.61
11	138	0.37	15	0.00	11.04	−*8.62*	9.20	1.05
18	124	−0.21	14	−0.46	11.64	−*6.98*	8.86	0.49
21	163	1.40	16	0.46	12.18	−*5.49*	10.19	*2.69*
26	145	0.66	13	−0.93	15.07	*2.43*	11.15	*4.29*
MYB42UTR								
28	92	−1.52	12	−1.39	14.00	−0.50	7.67	−1.49
6	109	−0.82	15	0.00	10.97	−*8.81*	7.27	−*2.15*
32	137	0.33	14	−0.46	16.10	*5.26*	9.79	*2.02*
30	71	−*2.39*	11	−1.85	12.56	−*4.45*	6.45	−*3.5*
15	147	0.74	15	0.00	16.34	*5.91*	9.80	*2.05*
26	174	1.85	18	1.39	14.65	1.28	9.67	1.83
16	162	1.36	17	0.93	13.41	−*2.12*	9.53	1.6

Overall averages for control (*n* = 3 individual plants) measurements are presented with standard deviation. *Z* scores represent the number of standard deviations that each MYB plant is from the control average, with *z* scores greater than 2 or −2 highlighted in italicized font. As phenotypic measurements could only be made once per transgenic plant, the number of standard deviations (*z* scores) for each MYB plant measurement were calculated against the transgenic controls. Plants are listed in ascending order of total lignin content

Most of the MYB ORF and UTR plants had smaller internode diameters. Of the MYB31ORF lines, except MYB31ORF 11, all other lines had significantly smaller diameter than the control plants. Among the MYB31UTR lines, MYB31UTR 7 had significantly larger diameter than the control plants (Table 1). The majority of the MYB31ORF lines had significantly longer internodes, whereas four lines of MYB31UTR had increased internode length. Of the MYB42 plants, two had larger internode diameters and four had smaller diameters. Similar results were seen for internode length, as four MYB42 plants had increased internode length and two had decreased length (Table 1).

### Quantitative PCR analysis of lignin biosynthetic pathway genes in *MYB*-expressing sugarcane

**Fig. 1:**
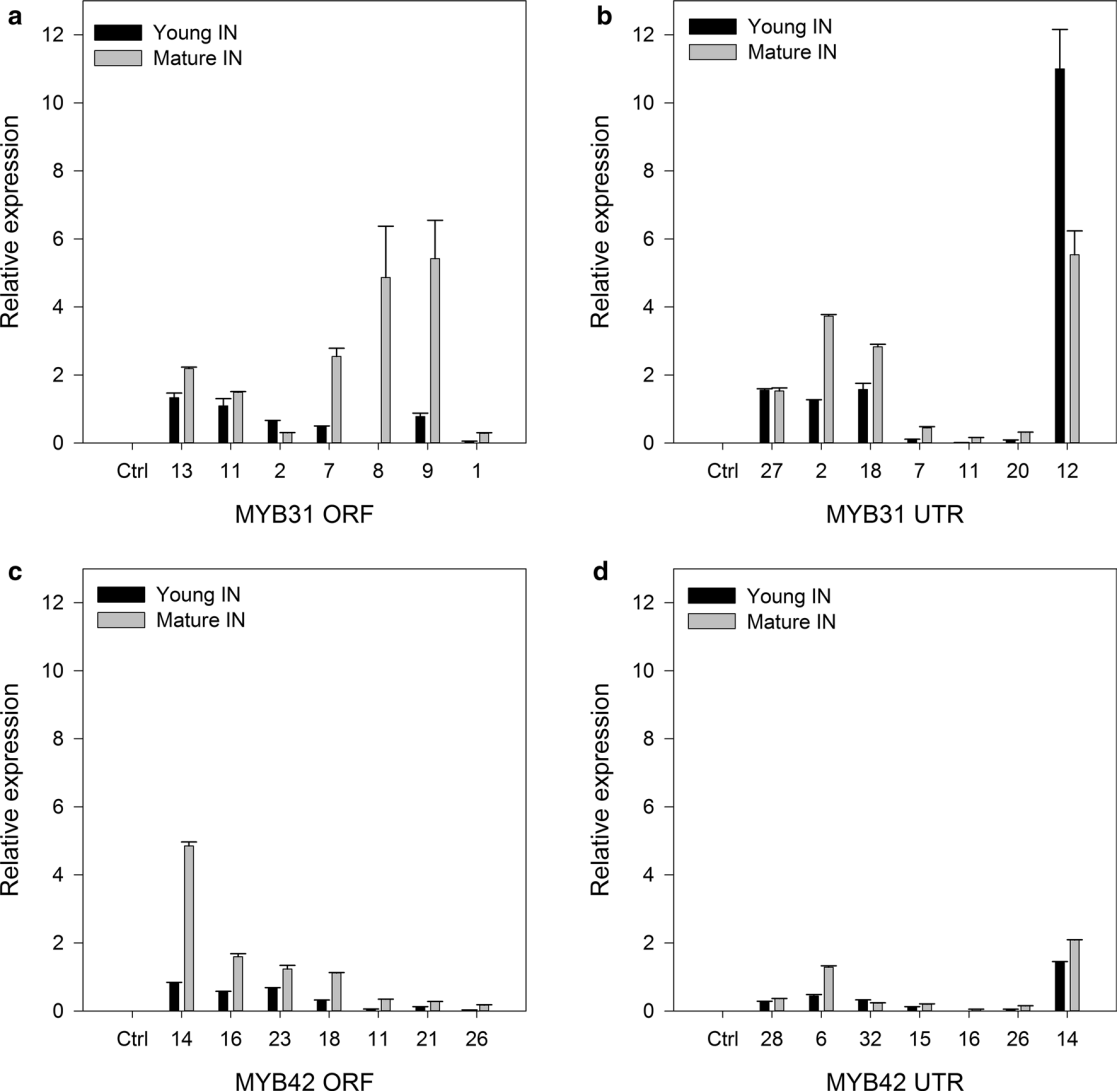
Quantitative expression of MYB32 and MYB42 in transgenic sugarcane. Quantitative PCR ΔCt values showing standard error of the mean of **a** MYB31ORF, **b** MYB31UTR, **c** MYB42ORF and **d** MYB42UTR expression in the MYB-transformed sugarcane plants after qPCR analysis of young and maturing internode tissues post-harvest. Each sample underwent qPCR in triplicate. Plants are listed in ascending total lignin content for each line. Control *n* = 3

**Fig. 2:**
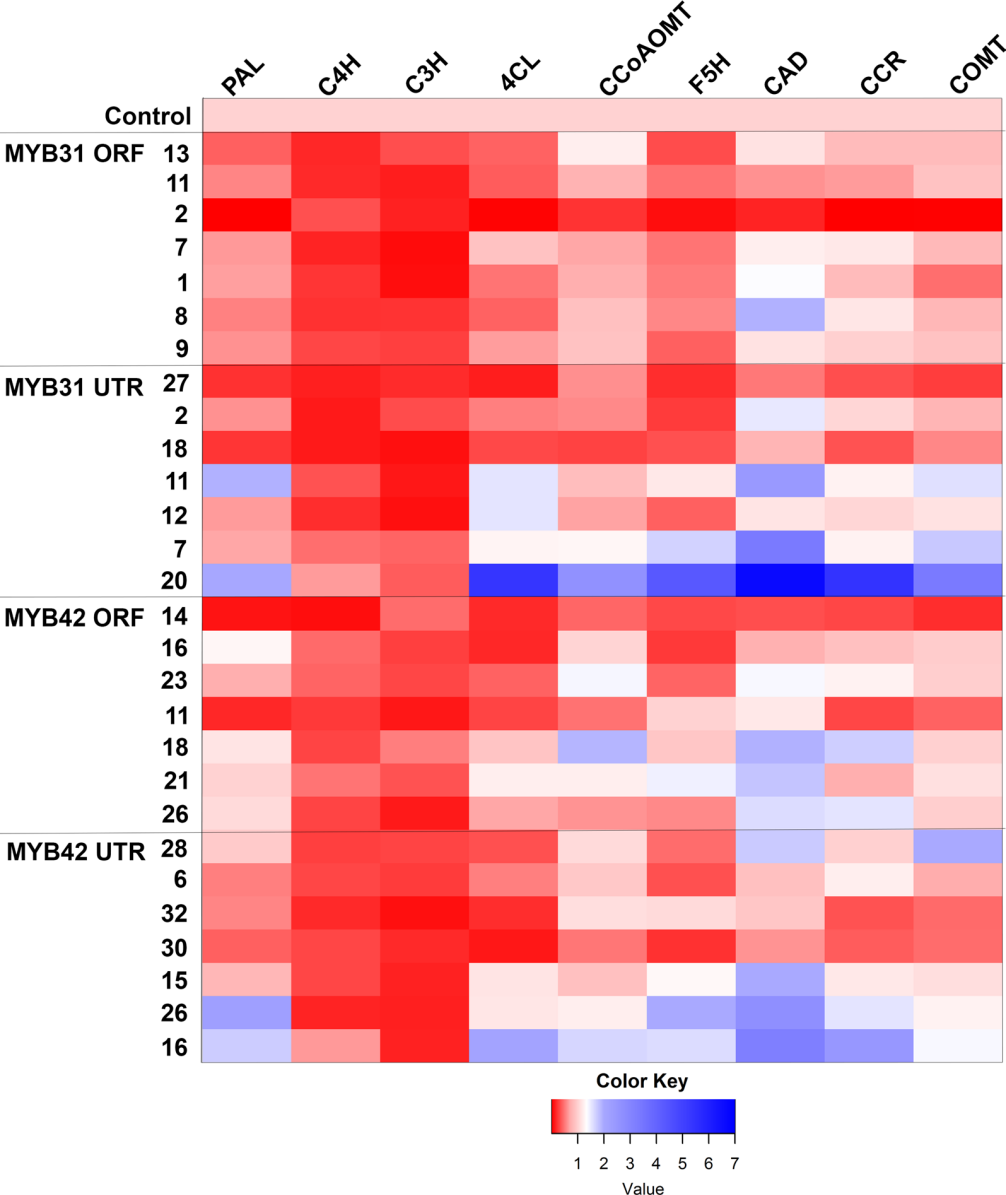
Quantitative PCR value heatmap of lignin biosynthetic genes in maturing internodes. Quantitative PCR ΔCt values of lignin biosynthesis genes in sugarcane expressing ZmMYB31 or ZmMYB42 normalized against GFP controls. Control *n* = 3. QPCR analysis of all tissue types and statistical analysis are displayed in Additional file 3

Quantitative PCR analysis was performed on cDNA synthesized from leaf and young and maturing sugarcane internodes for each transgenic plant along with corresponding GFP transgenic controls. The plants were initially analyzed for expression of the transgenes in the leaves (prior to being transferred to the greenhouse) to confirm the presence of the transgene. Subsequently, qPCR was performed on cDNA synthesized from young and maturing sugarcane internodes (following 9 months of greenhouse growth) for each control and transgenic plant. The majority of MYB31 and MYB42 plants continued to express the *MYB* transgene in both young and maturing internode tissues (Fig. 1; see Additional file 2), although the levels of expression for some plants, MYB31UTR11 and MYB42UTR16 for example, were very low. Of the plants with no detectable *MYB* expression, it appears that this was only in young tissue and expression was detected in the maturing tissue of the same plants. In general, there also appeared to be a trend of increased *MYB* transgene expression in maturing tissue when compared with young tissue in all lines. No transgene expression was detected in any tissue of any GFP control plant. After confirming the *MYB* transgene expression level, the expression levels of lignin biosynthetic genes were quantified by qPCR from the same cDNA samples (Fig. 2). See Additional file 3 for qPCR of all lines and statistical analysis.

*C4H* was the only gene significantly downregulated in all the events of MYB31 and MYB42 in the young internodes. In MYB31ORF plants, most of the lignin biosynthetic genes were significantly downregulated in the leaf, whereas in MYB31UTR plants, only *C3H* and *COMT* were significantly downregulated in leaf tissue. In young internodes, *C3H*, *4CL*, *F5H* and *COMT* were significantly downregulated in MYB31ORF, whereas in MYB32UTR only *C3H* was significantly different. In maturing internodes, *C3H* and *F5H* were significantly decreased in MYB31ORF. In MYB42 plants, MYB42ORF downregulated *C3H*, *F5H* and *4CL* in young internodes of most plants, whereas MYB42UTR downregulated only *C3H* in young internodes. MYB42 also upregulated *PAL* and *CAD* gene expression in internodes.

The overall trends suggest that *ZmMYB31* downregulated more genes than *ZmMYB42* when constitutively expressed in sugarcane (Fig. 2). See Additional file 3 for data on all lines and statistical analysis. Plants expressing *MYB31* had a downregulatory pattern which is spread across genes that are both early and late in the lignin biosynthesis pathway, whereas *MYB42* expression appeared to downregulate the early pathway genes, with the exception of *PAL*, more than the later pathway genes (Fig. 2; see Additional file 3). In common, both *MYB* genes downregulated *C4H* and exhibited an increase in *PAL* and *CAD* expression. Although not seen in MYB31 plants, MYB42 plants also showed increases in *CCoAOMT*, *CCR* and *COMT* expression. As well as downregulating more genes of the lignin biosynthetic pathway overall, *MYB31* also appeared to be more consistent in gene regulation across the different tissue types when compared with *MYB42* (see Additional file 3). The lignin gene expression results for MYB31 aligned with the published results more closely than the MYB42 expression results.

### Cell wall compositional analysis of *MYB* transgenic sugarcane

**Fig. 3:**
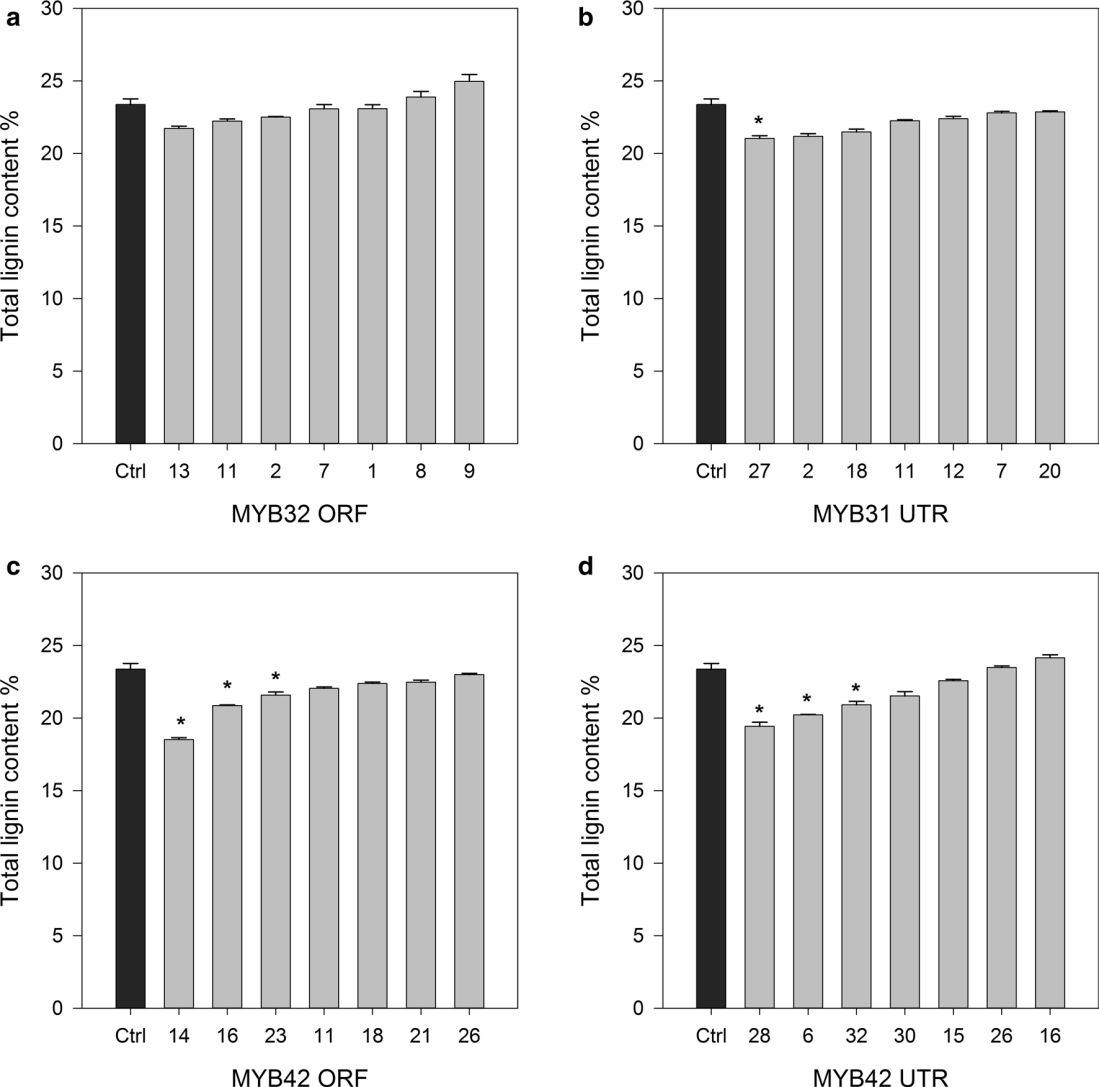
Total lignin content (%) of MYB32 and MYB42 transgenic sugarcane. Lignin content was quantified by a modified acid hydrolysis method. Acid-soluble lignin was determined by spectrophotometry and acid-insoluble lignin was measured gravimetrically. Acid-soluble lignin and acid-insoluble lignin were summed to determine total lignin content. **a** MYB31ORF, **b** MYB31UTR, **c** MYB42ORF and **d** MYB42UTR. Samples significantly different from the GFP controls after ANOVA followed by LSD test, *p* = 0.05, are shown with *asterisk*. Control *n* = 3. Data on all lines and statistical analysis are displayed in Additional file 4

**Fig. 4:**
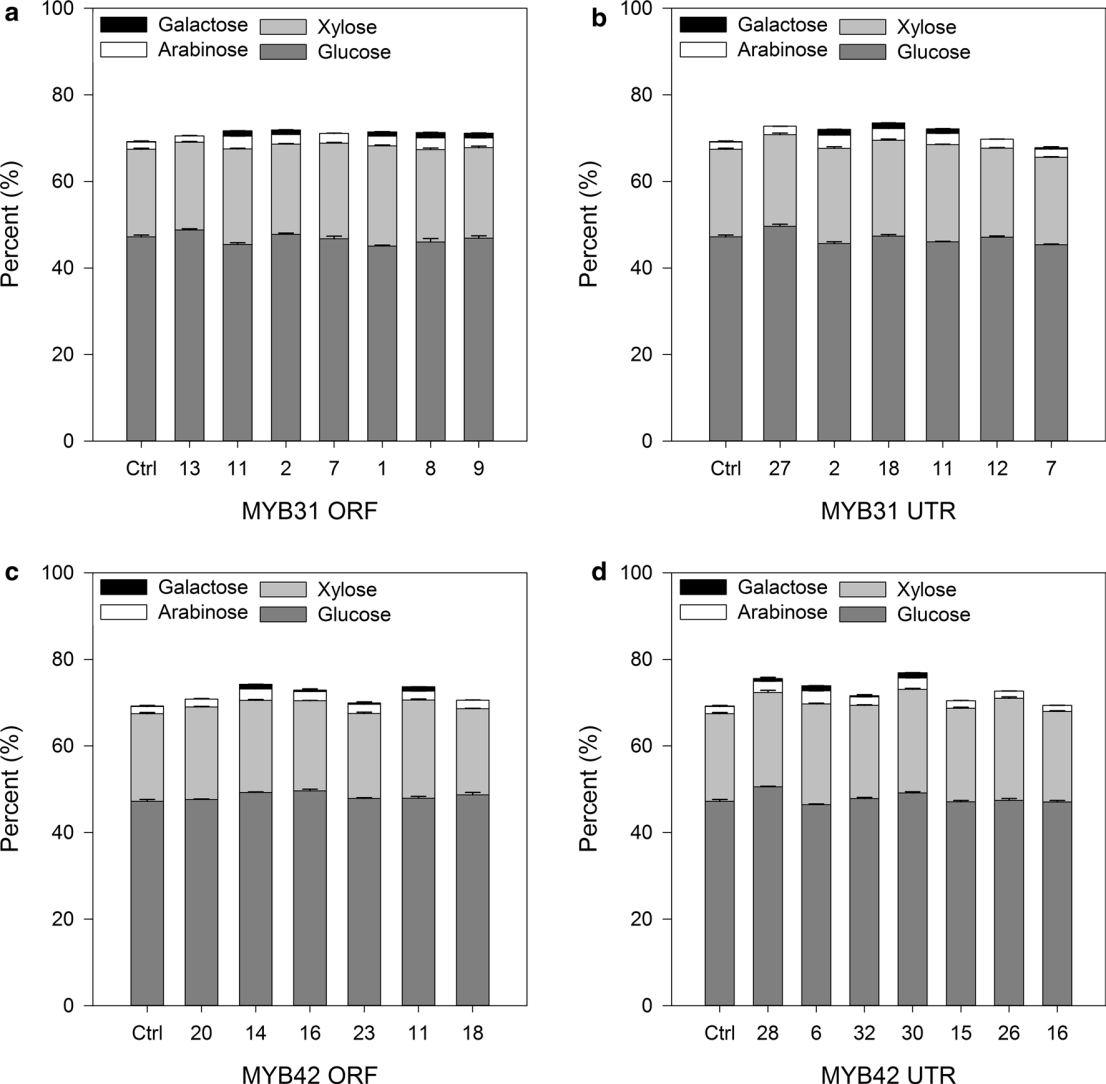
Total carbohydrate content (%) of MYB32 and MYB42 transgenic sugarcane. Cellulose and hemicelluloses were quantified by a modified acid hydrolysis method as described. **a** MYB31ORF, **b** MYB31UTR, **c** MYB42ORF and **d** MYB42UTR. The percentage of each component of the total composition is shown with the standard error of the mean. Data on all lines and statistical analysis are displayed in Additional file 4

Cell wall compositional analysis was performed to determine acid-soluble and acid-insoluble lignin content, as well as cellulose (glucose) and hemicellulose (xylose, galactose and arabinose) content. Of the *MYB31*-expressing sugarcane lines, only MYB31UTR 27 showed a significant decrease in total lignin content (Fig. 3; Additional file 4). This same plant also had significantly decreased acid-soluble lignin while having significantly increased glucose and xylose (Fig. 4; Additional file 4). Two other MYB31UTR plants showed a significant decrease in acid-insoluble lignin and a significant increase in xylose (see Additional file 4). Approximately, half the MYB31 plants (ORF and UTR) had significantly increased xylose and galactose (Fig. 4; see Additional file 4). Overall, *MYB31* expression appeared to have little impact on lignin and glucose levels, but increased the synthesis of structural hemicelluloses. There were no changes in ash content in *MYB31*-expressing lines.

Six sugarcane plants expressing *MYB42* (three ORF plants and three UTR plants) showed a significant decrease in lignin content (Fig. 3). This was the result of significant decreases in acid-insoluble lignin (see Additional file 4) as there were no significant changes to the acid-soluble component. Of these six plants, two had significant increases in glucose content (Fig. 4; see Additional file 4). There were significant increases in xylose, galactose and arabinose contents, but these increases were not specific to lignin-reduced plants. Significant decreases in structural carbohydrate contents were not found in any MYB42 plants. Only one of the fully characterized MYB42 lines (42ORF 14) had a significant increase in ash content. Both *MYB* genes appeared to have little effect on glucose content of plants, but the expression of *MYB31* had a more consistent effect on hemicellulose components.

### Enzymatic hydrolysis of MYB and control bagasse samples

**Fig. 5:**
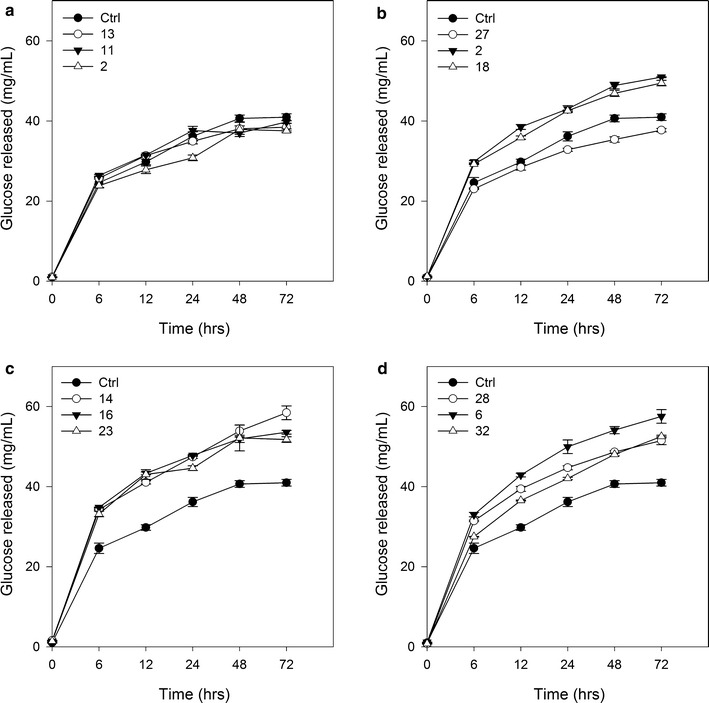
Enzymatic hydrolysis of MYB32 and MYB42 transgenic sugarcane. Total glucose concentration in enzymatic hydrolysis solution (mg/mL) per gram (g) of bagasse showing standard error of the mean measured at six time points over an incubation period of 72 h for **a** MYB31ORF plants, **b** MYB31UTR plants, **c** MYB42ORF, and **d** MYB42UTR using values from Additional file 5. Control *n* = 3

The three MYB plants per line with the largest decrease in lignin composition were selected to undergo enzymatic hydrolysis (Fig. 5). Hydrolysis was carried out for 72 h with sampling at six time points. When compared with GFP controls, only two of the six *MYB31*-expressing plants showed a significant increase in glucose release after 72 h, whereas all six *MYB42*-expressing plants released significantly more glucose than the GFP controls (Fig. 5; see Additional file 5).

Four MYB42 plants showed significantly higher rates of glucose conversion as early as the 6-h time point and continued to release significantly more glucose at each of the later time points (Fig. 5). The plants with significantly more glucose released after 72 h all showed signs of increased rate of glucose conversion by the 12-h time point (see Additional file 5). Significantly higher glucose released at earlier time points by the MYB sugarcane is indicative of an increased rate of cellulose conversion to glucose.

### Cellulose crystallinity index and sucrose content of *MYB* bagasse

The tissue requirements for pretreatment and enzymatic hydrolysis left enough bagasse for only two GFP control plants and five MYB plants to undergo determination of cellulose crystallinity index. Statistical analysis was not performed as the limited number of samples would not provide reliable results. The ranges of crystallinity were 49.03–51.49 % for control plants and 45.56–47.50 % for MYB-expressing plants (see Additional file 6).

**Fig. 6:**
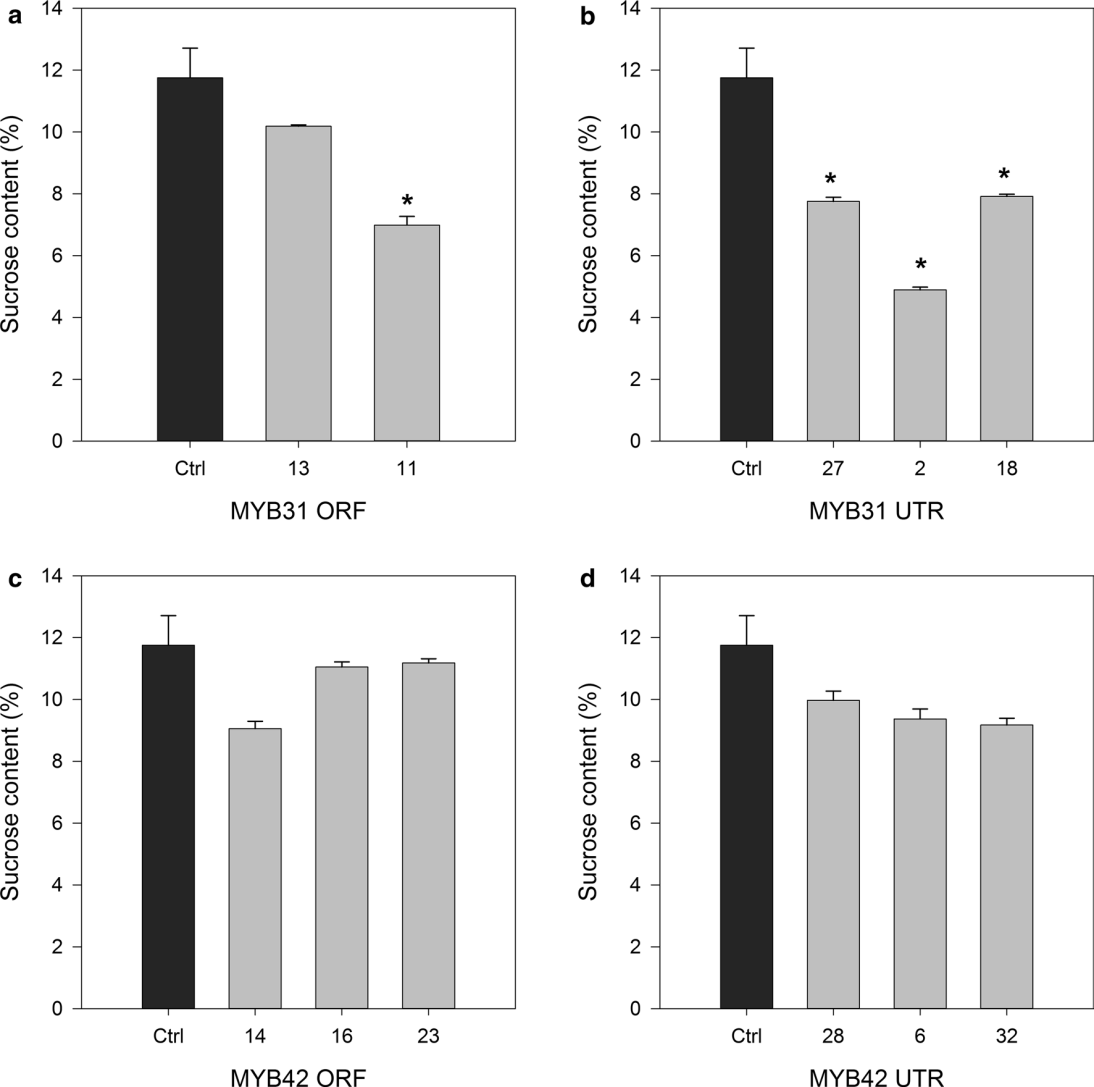
Sucrose content of extracted juice of transgenic sugarcane plants. Sucrose content (%/fresh weight) of extracted juice of transgenic sugarcane plants selected for enzymatic hydrolysis (showing standard error of the mean). **a** MYB31 ORF, **b** MYB31 UTR, **c** MYB42 ORF and **d** MYB42 UTR. An *asterisk* indicates a significant difference to GFP controls after ANOVA followed by LSD test, *p* = 0.05. GFP *n* = 3

Plants that underwent enzymatic hydrolysis were also assessed for sucrose content of the extracted juice (Fig. 6) to determine if changes in lignin content or structure had affected juice composition and quantity. Overall, only MYB31 plants showed a significant decrease in sucrose content when compared against the GFP control plants (Fig. 6) and MYB42 plants were comparable to controls. Glucose and fructose were not detected in any of the samples. MYB31ORF 2 did not have sufficient tissue for juice analysis.

### Lignin composition and structure

**Fig. 7:**
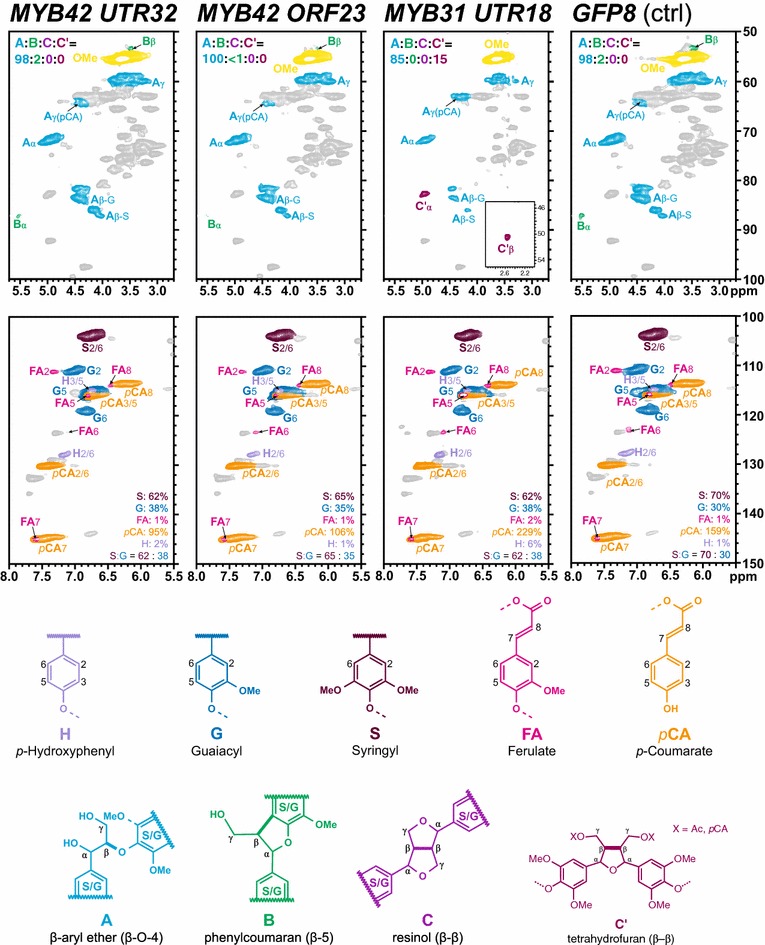
Partial 2D HSQC NMR spectra. Enzymatic lignins from the two MYB42 and the one MYB31 transgenic sugarcane bagasse, along with the GFP control in DMSO-d_6_/pyridine-d_5_ (4:1). The *top row* shows the aliphatic region, with *color*-*coded* assignments of the main lignin to the same-colored corresponding structures. The *bottom row* shows the aromatic and double-bond region showing the lignin polymer H, G, and S structural units along with ferulates (mainly from feruloylated arabinoxylan polysaccharides) and *p*-coumarates (on both lignin and polysaccharides); again, contour coloration matches that of the structures shown. The aromatic unit quantification values are from volume integration, on an S + G = 100 % basis; accuracy is generally good for the H:G:S units, but *p*-coumarate and ferulate end groups are severely overestimated, so their integrals should be used only for comparative purposes [17]

Samples with enough tissue remaining underwent NMR analysis to determine their lignin monomer compositions and altered structural features (Fig. 7). GFP controls had an S:G of 70:30, whereas the MYB lines all had small reductions in S:G. Ferulate (that is essentially all from ferulate on arabinoxylan) levels were all low (~1–2 % on an S + G = 100 % basis) across all lines, but *p*-coumarate (that is from both its acylation of arabinoxylans and of lignin) varied significantly with the MYB42 lines having lower levels than the control and the MYB31 line higher; the integrals of these end groups (Fig. 7) significantly overestimate the actual levels on a lignin basis in HSQC experiments [17], but the relative levels are indicative of the change. Changes in the levels of the major side chain structures **A**, **B**, **C** and **C′** (Fig. 7) are also evident. Particularly intriguing (see the Discussion) is the appearance of structures **C′** in the MYB31 line, indicating that its lignin chain initiation is different from the other lines, including the GFP control.

## Discussion

*ZmMYB31* and *ZmMYB42* belong to the R2R3-MYB superfamily of MYB transcription factors that are known to be involved in the regulation of the lignin biosynthetic pathway [12]. The R2R3 domains of MYB transcription factors bind to conserved AC elements within the promoters of lignin biosynthetic genes, allowing for regulation of gene expression levels [11, 18–20]. The cloned sequences of *ZmMYB31* and *ZmMYB42* were 99 and 98 % identical to the sequences published by Fornale et al. [12]. The R2R3 domains of the cloned sequences were 100 % identical to the published sequences. The differences are attributed to the varietal differences in corn used for cloning purposes and did not appear to negatively affect the expression of the *MYB* genes (Fig. 1; see Additional file 1) or their overall ability to downregulate the expression of genes within the lignin biosynthetic pathway (Fig. 2; see Additional file 2). Inclusion of UTR sequences has been shown to improve gene expression [21–25]. Although the constructs containing UTR sequences for both *MYB31* and *MYB42* produced more PCR- and qPCR-positive plants than their ORF counterpart, retaining or omitting the UTR sequences did not appear to affect the level of downregulation of lignin biosynthetic genes, deposition of cell wall components, juice sucrose levels, or plant phenotype in plants expressing MYB31 or MYB42. All MYB42ORF and MYB42UTR plants analyzed released significantly more glucose, indicating that the UTR sequences did not contribute any additional benefits to the enzymatic hydrolysis of bagasse. This could be due to the non-native maize promoter and UTR used here in sugarcane and the UTR effect being species specific [26]. However, including the UTR sequences did result in an increase in *MYB31*-expressing plants that released significantly more glucose after enzymatic hydrolysis.

*Zm*MYB31 and *Zm*MYB42 are known to downregulate multiple genes within the lignin biosynthetic pathway [10–12], and this downregulatory control was also observed in this study. MYB31 appears to downregulate all genes analyzed except *CAD*, whereas MYB42’s regulatory effects are more subtle. Although approximately half of the genes analyzed appear to be downregulated by MYB42, half appear to be upregulated. The genes downregulated by MYB42 generally appear earlier in the lignin biosynthetic pathway, and the genes upregulated function later in the pathway. The downregulatory effects of MYB31 were more consistent across the three tissue types analyzed than MYB42. These results suggested that MYB31 would reduce lignin content in sugarcane bagasse to a greater extent than MYB42.

Previous research showed a strong correlation between reduction of lignin gene expression by *MYB* genes and reduction in lignin content [10, 11, 14, 27, 28]. Of the 14 *MYB31*-expressing sugarcane lines analyzed, only MYB31UTR 27 had a significant decrease in total lignin, by 10 %. Lines MYB31UTR 2 and MYB31UTR 18 had 13 % reductions in acid-insoluble lignin but not total lignin. This is far from the eightfold [12] and 70 % [11] decreases in lignin content previously reported in Arabidopsis after the transgenic expression of *MYB31*. This difference may simply be due to the use of different promoters and terminators. However, it is more likely due to the large and complex polyploid sugarcane genome [29] that increases the likelihood of the existence of gene homologs. It is possible that MYB31 was only able to downregulate a number of these potential homologs in the current study, with the decreased expression compensated by unregulated homologs, thus reducing the overall regulatory effect of MYB31 on lignin biosynthesis. In addition, copy number was not assessed in these lines and can play a role in effect variability [30].

As is increasingly realized, pathways in general and the lignin biosynthetic pathway in particular differ significantly between monocots and dicots [31]. The side chain region of the spectra (Fig. 7, top row) showed some intriguing features. First is a feature even stranger than that recently commented on in maize [32]. The GFP control, but also the MYB42 lines, that were all S–G lignins with similar S:G, had abundant β-ether units **A** and small amounts of phenylcoumarans **B**; as noted in maize, there were imperceptible levels of the resinol units **C** that in dicots with similar S:G are at significant levels (5–10 %), derive from sinapyl alcohol dimerization, and represent the stating points of lignin polymerization. These sugarcane lines, therefore, appear to have lignin chains that are not initiated by monolignol dimerization. As these samples have little-to-no tricin, a flavone that was recently discovered in most monocots that nucleates lignin chain formation [31–33], the only discernible way that chains can initiate is from the ferulates (and diferulates) on arabinoxylans, as reviewed recently [34]. Even more intriguingly, the MYB31 line, with only slightly lower S:G than the control, exhibits the very clear presence, not of resinol units **C**, but of analogous units **C′** that derive from dimerization of acylated monolignols, primarily sinapyl *p*-coumarate, as in wild-type maize [34]. Clearly, these units are chain initiators in this MYB31 line, but not (to any significant degree) in the other lines. In another report on sugarcane bagasse NMR spectra, the whole cell walls appeared similarly devoid of β–β-coupling products (**C** and **C′**), but both were revealed at low levels in the spectra of isolated Björkman lignins [35]. Our enzyme lignins here represent essentially all of the lignin in the sample, but have a higher level of residual polysaccharides than purified Björkman lignins. We offer no explanations for the intriguing observations noted here between the lignins in sugarcane vs maize, but simply stress that these add to the contention that lignification in monocots is significantly different from that in dicots and that mysteries regarding the process and the structural features of the polymer remain.

Approximately, half of the *MYB31*-expressing sugarcane plants (ORF and UTR) had significantly increased xylose and galactose, whereas Fornale et al. [11] reported no changes in the structural carbohydrate composition of lignin-reduced Arabidopsis. Within sugarcane, *Zm*MYB31 expression appears to have little impact on lignin and glucose levels, but increases the synthesis of hemicelluloses.

Six sugarcane plants expressing *MYB42* (three ORF plants and three UTR plants) showed a significant decrease in total lignin content of 8–21 % (Fig. 3), whereas previous research found the Arabidopsis plants expressing *MYB42* had greater reductions in lignin content, of 2.1-fold [12] and 60 % [10] in different studies. Two sugarcane events had significant increases in glucose content, whereas no changes in cellulose content were found in *MYB42*-expressing Arabidopsis [10]. Hemicelluloses were increased in Arabidopsis [10] as well as in several *MYB42*-expressing sugarcane plants. These changes in carbohydrate content did not correlate with changes in lignin content.

These results suggest that, within sugarcane, MYB42 affects the deposition rates of lignin, whereas MYB31 increases the hemicellulose content within secondary cell walls, but may have little effect on lignin production. The finding that MYB31 downregulated the expression levels of the lignin biosynthetic pathway genes to a greater extent than MYB42, but lignin deposition was more influenced by MYB42 than MYB31, suggests that gene transcription levels may not necessarily reflect translation levels of lignin biosynthetic genes. It is possible that the genes with higher expression levels in young internode tissue have gene products that may persist during tissue maturation, allowing for continued lignin biosynthesis without the need for continued high expression of these genes. Although the genes of the lignin biosynthetic pathway have been well characterized, a full understanding of metabolic flux through the pathway remains to be established, making it difficult to predict the outcomes of modifying gene expression levels. Assaying the enzymatic activity of translated lignin biosynthetic genes, or the concentration of enzyme product throughout the stem, would provide a clearer picture of the regulatory effects of *Zm*MYB31 and *Zm*MYB42 expression over these genes.

Reduced lignin content in sugarcane has previously led to improved saccharification [13, 36–39] as has the expression of MYB31 and MYB42 in Arabidopsis [10, 11]. The expression of *Pv*MYB4 in transgenic switchgrass is the only report of a monocot species having improved saccharification resulting from the expression of an *MYB* gene [14, 15]. This is the first report of the expression of *MYB31* and *MYB42* in a monocot, and the first report of *MYB* transcription factors being expressed in sugarcane to alter lignin biosynthesis for improved biofuel production. In this study, the three plants with the least amount of total lignin from each line were assessed for enzymatic hydrolysis performance against controls over a period of 72 h. At 72 h, the MYB42 lines performed better than the MYB31 lines and GFP controls. These plants also had a faster rate of glucose conversion than controls. The compositional analysis showed that all six of these plants had significant reductions in total lignin as a result of reductions in acid-insoluble lignin. In contrast to these results, no MYB31ORF plants and only two MYB31UTR plants had higher levels of glucose released than controls at 72 h. The MYB31ORF results are unsurprising, as no MYB31ORF plants had reductions in lignin (see Additional file 4). However, MYB31UTR 27 had significantly less total lignin, but did not release significantly more glucose, whereas MYB31UTR 2 and MYB31UTR 18 did not have significantly less total lignin, but did release significantly more glucose; the latter observation is likely due to the significant reductions in acid-insoluble lignin in these two plants, or maybe due to changes in lignin composition and structure, as MYB31UTR 18 was shown to have a similar S:G ratio to the other MYB lines (and slightly lower than the control) and a lot higher level of *p*-coumarate (although it is not clear whether this is on the lignin or the hemicelluloses) relative to GFP controls (Fig. 7). The finding that all MYB31 and MYB42 plants with significant reductions in acid-insoluble lignin content performed significantly better than transgenic controls after enzymatic hydrolysis suggests that this cell wall component is a primary influencing factor on enzymatic hydrolysis performance. Decreases in acid-insoluble lignin have also been reported alongside improvements in saccharification in switchgrass [40], maize [41] and Arabidopsis [10, 11].

The current finding that MYB42 lines outperformed MYB31 lines during enzymatic hydrolysis is supported by previous research. In transgenic Arabidopsis, the expression of *MYB42* improved the enzymatic hydrolysis by 68 % [10] compared with an increase of 14 % after the expression of *MYB31* [11]. Due to limited bagasse available for analysis, the enzymatic hydrolysis section of this study utilized one pretreatment condition (1 % w/w sulfuric acid) and subsequently a single digestive enzyme cocktail mix at one concentration (six FPU Accellerase 1500 with the addition of β-glucosidase). Of the different pretreatments available, mild acid pretreatment was selected, as this would not change the lignin content of bagasse and disrupts the hemicellulose content of lignocellulosic biomass to expose the cellulose to hydrolytic enzymes [42, 43], thus better elucidating any digestive differences between bagasse samples with differences in lignin content. A low FPU concentration was used to again better elucidate any differences in bagasse digestibility. It would be expected that the use of different pretreatment conditions or cocktail mixes or concentrations would produce different results. For example, if an alkaline pretreatment was used, which removes lignin as opposed to hemicellulose [43], it would be expected that transgenics and wild types would release similar amounts of glucose as the glucose contents between controls and transgenics do not overly differ.

For transgenic sugarcane expressing MYB genes to be beneficial to, and accepted by, the sugarcane industry, it is important that any change in cell wall composition is not detrimental to the phenotype of the sugarcane or to the juice sucrose levels that are the cornerstone of the sugarcane industry. The sucrose content did not significantly decrease in MYB42 plants tested, even though these same plants had significantly reduced lignin content. These MYB42 plants also showed increased glucose release from the bagasse. It therefore appears to be possible to produce sucrose and improved biomass in a single plant. Overall, the majority of phenotypic differences were in internode diameter and length for both MYB31 and MYB42 plants, with few differences seen in plant height or total number of internodes. The differences in phenotypic measurements were not specific to plants with significantly reduced lignin contents or altered polysaccharide contents. Previous research has reported decreased growth rates and dwarfed phenotypes after MYB directed lignin reductions in poplar and Arabidopsis [44], tobacco [14, 27, 45] and switchgrass [14, 15]. Reduced height was also observed in Arabidopsis expressing *ZmMYB31* and *ZmMYB42* [10–12]. The absence of a strong detrimental phenotype in the current study may be due to the relatively low levels of *MYB* transgene expression. It is possible that alterations in lignin biosynthesis can also have an impact on a plants’ resistance to pests and pathogens [46]. This was not assessed in the MYB-overexpressing sugarcane, but would be a necessary assessment prior to commercial use.

Arabidopsis expressing MYB31 [11] and MYB42 [10] with reduced height had severe lignin reductions of up to 70 and 60 %, respectively. Vanholme et al. [47] suggested that more modest lignin reductions may not result in these detrimental phenotypes, as is supported by the current findings. Of the six MYB31 plants that underwent enzymatic hydrolysis, only one plant had a decrease in total lignin and two plants in acid-insoluble lignin of 10 and 13 % (see Additional file 4). This is better highlighted by the MYB42 plants in this study. The six MYB42 plants that underwent enzymatic hydrolysis had significant reductions in lignin content (Fig. 3; see Additional file 5), but were not different in height to controls (Table 1). The greatest lignin reduction was by 21 % in MYB42ORF 14, which is modest when compared with the 60–70 % reductions previously reported in detrimental phenotypes [10, 11] and may help explain why the MYB31 and MYB42 lignin-reduced plants in this research did not show any height differences. Some of these lignin-reduced plants did show differences in average internode diameter and length that may be attributed to the changes in cell wall production.

## Conclusions

Although *MYB31* expression resulted in the downregulation of many genes within the lignin biosynthetic pathway, this did not carry over to cell wall synthesis, as only a limited number of MYB31 sugarcane plants had reductions in lignin content. This limited the potential saccharification improvements of *MYB31* expression. Alternatively, *MYB42* expression in sugarcane more closely met the aims of this research, with all MYB42 plants analyzed by enzymatic hydrolysis having increased glucose release with no reduction to juice sucrose levels and minimal phenotypic effects. This research highlights *Zm*MYB42 as a transcription factor of interest for improving the production of second-generation bioethanol from sugarcane bagasse.

## Methods

### Cloning of *ZmMYB31* and *ZmMYB42*

RNA was extracted from combined maize leaf and stem tissues sourced from locally grown sweet corn and used for cDNA synthesis. Primers were designed from available sequence information to amplify *MYB31* and *MYB42* genes from maize cDNA (see Additional file 7). Two amplicons were cloned for each *MYB* gene to either include or exclude the adjacent sequence of the 5′ and 3′ untranslated regions (UTR) (see Additional file 1), as there is evidence that inclusion of UTR sequences may improve the expression [22]. ‘MYB UTR’ refers to DNA with the inclusion of untranslated regions and ‘MYB ORF’ refers to the open reading frame only. MYB31UTR and MYB42UTR had 128 and 64 bp of 5′ UTR and 251 and 32 bp of 3′ UTR, respectively, amplified. The resulting PCR products were cloned into the pGEM-T Easy Vector System. After sequence confirmation, fragments were subcloned into the *SmaI* site of an existing alkaline phosphatase-treated *Zm*Ubi-iUbi-nos/pBlueScript entry vector containing the maize Ubiquitin promoter (Ubi) and intron (iUbi) [48, 49] and the nopaline synthase (nos) terminator [50], resulting in four *Zm*Ubi-iUbi-*MYB*-nos/pBS constructs.

### Sugarcane transformation and regeneration of plants

Callus was co-bombarded with individual *Zm*Ubi-iUbi-*MYB*-nos/pBS constructs and *Zm*Ubi-iUbi-*nptII*-nos/pUC19. Transformation control callus was co-bombarded with 1 μg *Zm*Ubi-iUbi-GFP-nos/pUC19 constructs with 1 μg of *Zm*Ubi-iUbi-*nptII*-nos/pUC19. Regeneration of callus was carried out as previously described [51], after which individual events were transferred to growth chambers for continued development. The confirmed *Zm*Ubi-iUbi-*GFP*-nos plants served as transgenic controls.

Tissue culture plantlets selected for acclimatization were transferred to growth rooms under a 16 h photoperiod at 25 °C with watering every second day. When the plants reached approximately 30 cm in height, they were transferred to the greenhouse and grown in 4.5 L pots at 27 ± 3 °C under natural light. Plants were watered to saturation twice per week and fertilized with Aquasol (Yates) once per month with regular removal of tillers. Potted plants underwent periodic randomized position rotation within the glasshouse to minimize positional effects.

### Nucleic acid extraction and quantitative PCR

DNA was extracted from 2- to 4-week-old leaf tissue using the rapid release method [48] with the addition of a chloroform:isoamyl alcohol (24:1) wash step after the 95 °C incubation [49]. A 1:4 dilution of the supernatant was used for subsequent PCR reactions. RNA was extracted from all tissue samples using Tri Reagent (Sigma, NSW, Australia) following the manufacturer’s protocol using tissue ground under liquid nitrogen. Briefly, tissue was incubated at room temperature with Tri Reagent for 5 min before the addition of 200 µL chloroform. Tubes were vigorously mixed and incubated at room temperature for a further 5 min. The tubes were centrifuged (12,000*g*, 15 min, 4 °C) and the supernatant collected and mixed with an equal volume of isopropanol. After 10 min incubation at room temperature, the reactants were again centrifuged (12,000*g*, 10 min, 4 °C) and the RNA pellet subsequently washed in 75 % ethanol before a final centrifugation (7500*g*, 5 min, 4 °C). The supernatant was removed and the pellet dried for 10 min before being resuspended in 30 µL water and incubated at 60 °C for 10 min. The extracts were kept on ice and used for cDNA synthesis immediately or stored at −80 °C. Extracted RNA (1 µg) was digested with RQ1 RNase-free DNase (Promega, NSW, AUS) following the manufacturer’s methods with the 37 °C incubation being increased to 1 h. After digestion, the samples were either used immediately for cDNA synthesis or stored at −80 °C. Complementary DNA synthesized from leaf tissue was used to confirm the presence of the transgene by qPCR analysis [27, 52] with the addition of a chloroform:isoamyl alcohol (24:1) wash step after the 95 °C incubation [53]. A 1:4 dilution of the supernatant was used for subsequent PCR reactions. Plants confirmed to contain the *MYB* transgene cassette were sampled for RNA extraction, cDNA synthesis and qPCR quantification of *ZmMYB31* or *ZmMYB42* transcript level, as well as the expression of nine lignin biosynthesis pathway genes in the young and maturing internodes. Initially, cDNA was extracted from internode 1 (young tissue) and internode 7 (maturing tissue) for quantitative PCR (qPCR) analysis. Seven individual plants per MYB construct (28 MYB plants in total) were selected for this analysis based on these plants having the greatest number of lignin biosynthetic genes downregulated along with three GFP transgenic controls. See Additional files 7 and 8 for primers used for both end point and qPCR. Quantitative PCR was performed on all seven plants generated, but only three plants were selected for subsequent analysis.

### Harvest and line selection for analysis

Events harboring the *MYB* gene and having detectable levels of *MYB* gene expression were transferred to the glasshouse and grown for 9 months before being destructively harvested for analysis. All plants were watered to saturation 2 days before harvesting to avoid results being affected by any potential drought-related stress response. The plants were harvested between 10 am and 2 pm in a single session to minimize light- or circadian-related fluctuations in gene expression levels [54, 55] and occurred over four consecutive days. Before measuring and cutting, all leaf tissue and sheaths were removed, and the internodes were counted as per van Dillewijn [56]. The length of the stalk was measured (internode 1 to the final internode), the number of internodes was counted and the diameter of the third internode from the base was recorded using calipers. Analyses were performed in a predetermined order and used only harvested internode tissue.

Plants that were analyzed by qPCR in the internode tissues also underwent acid hydrolysis to quantify their cell wall composition. Enzymatic hydrolysis was performed on three plants per MYB line, selected based on having the lowest lignin contents after compositional analysis, and three GFP transgenic control plants over 72 h with sampling at six different time points. Quantification of juice sugar components was performed on the plants selected for enzymatic hydrolysis, where there was enough tissue remaining.

### Cell wall compositional analysis

After harvest, tissue for cell wall compositional analysis was prepared as per [57] using convection oven drying at 40 °C. Dried samples were milled with an IKA Labortechnik MFC mill (2 mm screen) and subjected to successive overnight Soxhlet washes with water and then ethanol to remove extractives [58]. Samples were again dried using convection oven drying at 40 °C and stored at room temperature. A sample of this prepared material was dried overnight in a convection oven set at 105 °C and used to determine the total solids of the bagasse [59].

Lignin, cellulose and hemicelluloses were quantified by a modified acid hydrolysis method [58] using 0.125 g of bagasse and 1.5 mL 72 % (w/w) sulfuric acid. Acid-soluble lignin was determined by spectrophotometry, and acid-insoluble lignin and ash content was measured gravimetrically [58]. Structural carbohydrates were analyzed using high-performance liquid chromatography (HPLC) [58]. A Waters e2695 Separations Module and Shodex SP-0810 sugar column (85 °C) with micro-guard de-ashing columns (BioRad) equipped with a Waters 2414 Refractive Index Detector was employed. Samples for HPLC analysis were neutralized by addition of CaCO_3_ (50 mg/mL).

### Pretreatment of bagasse for enzymatic hydrolysis

Finely ground bagasse (McCrone micronizing) was subjected to a mild pretreatment. This involved 1 % (w/w) sulfuric acid being added in a ratio of 10:1 with bagasse followed by autoclaving (130 °C for 30 min). Samples were then washed with water. Total solid content (%) [59] was determined before use for enzymatic hydrolysis. Non-pretreated bagasse samples were used as a control during enzymatic hydrolysis to confirm the effectiveness of pretreatment. Samples then underwent acid hydrolysis as described above [58] to quantify lignin, cellulose and hemicellulose contents of the pretreated samples.

### Enzymatic hydrolysis of bagasse

Enzymatic hydrolysis of transgenic and GFP control bagasse was performed using Accellerase 1500 (Genencor). Before use, the filter paper units (FPU) and protein concentrations of the enzyme solution were determined. The FPU activity of Accellerase 1500 was calculated with the methods by [60], using 50 mM citrate buffer, and was determined to be 46.8 FPU/mL. The protein content of Accellerase 1500 was determined using a Bradford assay [61] and was found to be 22.87 mg/mL. Bovine serum albumin was used to develop a protein standard curve.

Enzymatic hydrolysis was performed following published methods [62]. Ground bagasse samples were mixed with 50 mM sodium acetate + 0.02 % (w/v) sodium azide to a concentration of 1.3 % cellulose (w/v) and rotated overnight by a Suspension Mixer (Ratek) at 4 °C. A 2× enzyme master mix was prepared containing Accellerase 1500 and *Aspergillus niger* β-glucosidase (Megazyme) to ensure complete hydrolysis of cellobiose to glucose. The final reaction concentration of Accellerase 1500 was 6 FPU (2.93 µg/g cellulose) and β-glucosidase was 50 µg/g cellulose. A 100 µL aliquot of the bagasse suspension was mixed with 100 µL of the 2× enzyme master mix resulting in a final cellulose concentration of 0.65 % (w/v).

Both pretreated and non-treated control bagasse samples were digested in triplicate at 50 °C with rotation for 72 h with samples being taken at 0, 6, 12, 24, 48 and 72 h. Reactions were quenched in liquid nitrogen and stored at −80 °C. The glucose released in each sample was analyzed using a d-Glucose Assay (GOPOD Format) (Megazyme) following the manufacturer’s instructions.

### Determination of cellulose crystallinity index in bagasse

Finely ground bagasse was used to determine the cellulose crystallinity index following the methods of Segal et al. [63]. X-ray diffraction patterns of cellulose samples were recorded with a Bruker AXS D8 Advance X-ray diffractometer at room temperature from 10 to 40 °C using Cu/Kα_1_ irradiation (1.54 Å) at 40 kV and 40 mA. The scan speed was 15 s/step with a step size of 0.05. The crystallinity Index (CI) was obtained from the relationship between the intensity of the 002 peak for cellulose I (I_002_) and the minimum dip (I_am_) between the 002 and the 101 peaks using the equation: CI (%) = ((I_002_–I_am_)/I_002_) × 100, where I_002_ = intensity at 22.7 Å and I_am_ = 18 Å. The divergence slit and anti-scatter slit were 3.722°. The program XRD commander (Bruker) was used to collect and analyze the data from the diffractometer.

### Juice extraction and component quantification

Juice was hot water extracted from internodes ground under liquid nitrogen following the methods of Inman-Bamber et al. [64]. Juice samples were diluted according to ICUMSA method GS7/8/4-24 using lactose as an internal standard and quantified using high-performance ion chromatography (HPIC). A Waters e2695 Separations Module and Dionex CarboPac PA1 HPLC column with guard column (27 °C) (Thermo-Fisher Scientific) equipped with a Waters 2465 Electrochemical detector was employed.

### Preparation of samples for NMR analysis

The whole plant cell wall gel-state NMR samples were prepared as previously described [65]. In brief, the dried cell wall sample was pre-ground for 30 s in a Retsch MM400 mixer mill at 30 Hz, using zirconium dioxide (ZrO_2_) vessels (10 mL) containing ZrO_2_ ball bearings (2 × 10 mm). The cell walls were extracted with distilled water (ultrasonication, 1 h, three times) and 80 % ethanol (ultrasonication, 1 h, three times). The cell walls were dried and finely milled using a Fritsch planetary micro mill PULVERISETTE 7 (Germany) at 600 rpm with zirconium dioxide vessels (20 mL) containing with zirconium dioxide ball bearings (10 mm × 10). Each sample (200 mg) was ground for a total of 2 h 40 min (interval: 10 min, break: 5 min, repeated 11×). The cell walls were suspended in sodium acetate buffer (45 mL, pH 5.0), inoculated with Cellulysin™ (100 mg, Calbiochem, USA) and incubated at 35 °C for 72 h. The solids were pelleted by centrifugation (20 min, 8000 rpm). The pelleted material was collected and treated with Cellulysin a second time. After the second cellulose treatment the pelleted material was washed three times with RO water (45 mL, ultrasonication 10 min, pelleted by centrifugation). After lyophilization, the obtained enzymatic lignin (30 mg) was dissolved in 0.8 mL DMSO-d_6_/pyridine-d_5_ (4:1, v/v) and subjected to NMR characterization.

### NMR analysis of lignin monomer composition and structure

NMR spectra were recorded at 25 °C on a Bruker Biospin (Billerica, MA) AVANCE 700 MHz spectrometer fitted with a cryogenically cooled 5 mm QCI gradient probe with inverse geometry (proton coil closest to the sample). Bruker’s Topspin 3.1 (Mac) software was used to process the spectra. The central solvent peak was used as the internal reference (δ_C_/δ_H_: DMSO-d_6_, 39.5/2.95) HSQC NMR experiments for the whole plant cell wall gel-state samples were performed as previously described [17, 65–67].

### Statistical analysis

Statistical analysis involved either a two-tailed *t* test assuming unequal variance, *p* = 0.05, or a one-way ANOVA with Tukey post hoc analysis or LSD to determine letter groupings, *p* = 0.05, as appropriate, comparing transgenic plants to transgenic controls. As phenotypic measurements could only be made once per transgenic plant, the number of standard deviations (*z* scores) for each MYB plant measurement were calculated against the GFP transgenic controls. Measurements were considered different to controls if a *z* score was greater than 2 or −2.

## Additional files

10.1186/s13068-016-0559-1 Nucleotide and amino acid alignments of MYB31 and MYB42. Alignments of published (Fornalé et al. 2006) and cloned nucleotide and amino acid sequences (ORF and UTR) for ZmMYB31 (NM_001112479) and ZmMYB42 (NM_001112539). Alignments were made using the Kyoto University Bioinformatics Center website (http://www.genome.jp/tools-bin/clustalw) and amino acid translations were made using the ORF region of each MYB nucleotide sequences and Vector NTI software. The start and stop codons are underlined in nucleotide sequence alignments with the 5′ and 3′ UTR regions being upstream and downstream of the start and stop codons respectively. The R2 and R3 motifs in each sequence are underlined with light gray and dark grey shading respectively.
10.1186/s13068-016-0559-1 Normalized MYB genes ΔCt values. Mean ΔCt value for MYB31 and MYB42 genes. Values represent post-harvest expression results from young internode tissue and maturing internode tissue. SE: standard error of the mean. Control *n* = 3. Letter groupings were determined by ANOVA followed by LSD test.
10.1186/s13068-016-0559-1 Normalized lignin biosynthetic genes ΔCt values for MYB-expressing plants. Values normalized against average control ΔCt value for each gene. Values represent initial expression screening of leaf tissue and post-harvest expression results from young internode tissue and maturing internode tissue. NE: Normalized expression with standard error of the mean shown. Samples significantly different to the controls after ANOVA, *p* = 0.05, are shown in bold. Control *n* = 3. Plants are listed in ascending total lignin content for each line.
10.1186/s13068-016-0559-1 MYB-expressing sugarcane cell wall composition. The percentage of each component of the total composition is shown with the standard error of the mean. Samples significantly different to the controls after ANOVA followed by LSD test, *p* = 0.05, are shown in bold. Control *n* = 3. Plants are listed in ascending total lignin content for each line.
10.1186/s13068-016-0559-1 Glucose release by enzymatic hydrolysis. Glucose released into enzymatic hydrolysis solution (mg/mL) per gram (g) of bagasse measured at six time points over 72 h. The glucose released is shown with standard error of the mean. Samples significantly different to the control group after ANOVA followed by LSD test, *p* = 0.05, are shown in bold. Control *n* = 3.
10.1186/s13068-016-0559-1 Cellulose crystallinity index of MYB bagasse. CI was calculated from the height ratio between the intensity of the crystalline peak (I_002_–I_AM_) and total intensity (I_002_) after subtraction of the background signal.
10.1186/s13068-016-0559-1 Primers for *Zm*MYB cloning, genomic PCR and qPCR. Primers designed from GenBank accessions for PCR amplification of *Zm*MYB31 and *Zm*MYB42 genes from maize cDNA (1–4), for leaf gDNA screening from *Zm*MYB31 and *Zm*MYB42 gene cassettes (5–8) and qPCR quantification of MYB gene expression in young and maturing internodes (9–10).
10.1186/s13068-016-0559-1 Primers for qPCR of lignin biosynthetic genes.

## Abbreviations

4CL: 4-coumarate CoA ligase
ANOVA: analysis of variance
C3H: *p*-coumarate 3-hydroxylase
C4H: cinnamate 4-hydroxylase
CAD: cinnamyl alcohol dehydrogenase
CCoAOMT: caffeoyl-CoA *O*-methyltransferase
CCR: cinnamoyl CoA reductase
COMT: caffeic acid 3-*O*-methyl transferase
F5H: ferulate 5-hydroxylase
FPU: filter paper unit
G: guaiacyl lignin (unit)
GFP: green fluorescent protein
H: *p*-hydroxyphenyl lignin (unit)
HPIC: high-performance ion chromatography
HPLC: high-performance liquid chromatography
LSD: least significant difference
NOS: nopaline synthase
ORF: open reading frame
PAL: phenylalanine ammonia-lyase
PCR: polymerase chain reaction
qPCR: quantitative PCR
S: syringyl lignin (unit)
UBI: ubiquitin
UTR: untranslated region
