# Supplementary material for: Sugarcane transgenics expressing MYB transcription factors show improved glucose release
Source: Biotechnol Biofuels. 2016 Jul 15;9:143. doi: 10.1186/s13068-016-0559-1 (PMC4946106; doi:10.1186/s13068-016-0559-1)
Supplement: Supplementary file 1 — 10.1186/s13068-016-0559-1 Nucleotide and amino acid alignments of MYB31 and MYB42. Alignments of published (Fornalé et al. 2006) and cloned nucleotide and amino acid sequences (ORF and UTR) for ZmMYB31 (NM_001112479) and ZmMYB42 (NM_001112539). Alignments were made using the Kyoto University Bioinformatics Center website (http://www.genome.jp/tools-bin/clustalw) and amino acid translations were made using the ORF region of each MYB nucleotide sequences and Vector NTI software. The start and stop codons are underlined in nucleotide sequence alignments with the 5′ and 3′ UTR regions being upstream and downstream of the start and stop codons respectively. The R2 and R3 motifs in each sequence are underlined with light gray and dark grey shading respectively. [file 13068_2016_559_MOESM1_ESM.pdf]

Figure S1 Nucleotide and amino acid alignments of MYB31 and MYB42

S1a: MYB31 nucleotide sequence alignment

```
MYB31_ORF       ------------------------------------------------------------
MYB31_UTR       ------------ACAGCAGCAACAACAACAACAACCTCCACTGCCGCAACCCACCGAGAG
NM_001112479    GCATCGCACCTCACAGCAGCAGCAACAACAACAACCTCCACTGCCGCAACCCACCGAGAG
                            ********* **************************************

MYB31_ORF       ------------------------------------------------------------
MYB31_UTR       GCGAGACCGGCGGCGGCAAAAGGACGATACAAAAGCAGCCAGGGTTGCTGGCAACAGCGT
NM_001112479    GCGAGACCGGCGGCGGCAAAAGGACGATACAAAAGCAGCCAGGGTTGCTGGCAACAGCGT
                ************************************************************

MYB31_ORF       --------------------ATGGGGAGGTCGCCGTGCTGCGAGAAGGCGCACACCAACA
MYB31_UTR       CGGTCGCCCGCCCGCTCGCCATGGGGAGGTCGCCGTGCTGCGAGAAGGCGCACACCAACA
NM_001112479    CGGTCGCCCGCCCGTACGCCATGGGGAGGTCGCCGTGCTGCGAGAAGGCGCACACCAACA
                **************  ********************************************

MYB31_ORF       AGGGCGCGTGGACCAAGGAGGAGGACGAGCGCCTGGTCGCGCACATCAGGGCGCACGGCG
MYB31_UTR       AGGGCGCGTGGACCAAGGAGGAGGACGAGCGCCTGGTCGCGCACATCAGGGCGCACGGCG
NM_001112479    AGGGCGCGTGGACCAAGGAGGAGGACGAGCGCCTGGTCGCGCACATCAGGGCGCACGGCG
                ************************************************************

MYB31_ORF       AGGGGTGCTGGCGCTCGCTGCCCAAGGCCGCCGGCCTCCTGCGCTGCGGCAAGAGCTGCC
MYB31_UTR       AGGGGTGCTGGCGCTCGCTGCCCAAGGCCGCCGGCCTCCTGCGCTGCGGCAAGAGCTGCC
NM_001112479    AGGGGTGCTGGCGCTCGCTGCCCAAGGCCGCCGGCCTCCTGCGCTGCGGCAAGAGCTGCC
                ************************************************************

MYB31_ORF       GCCTCCGCTGGATCAACTACCTCCGCCCCGACCTCAAGCGCGGCAACTTCACGGAGGAGG
MYB31_UTR       GCCTCCGCTGGATCAACTACCTCCGCCCCGACCTCAAGCGCGGCAACTTCACGGAGGAGG
NM_001112479    GCCTCCGCTGGATCAACTACCTCCGCCCCGACCTCAAGCGCGGCAACTTCACGGAGGAAG
                ********************************************************** *

MYB31_ORF       AGGACGAGCTCATCGTCAAGCTGCACAGCGTCCTCGGCAACAAGTGGTCCCTGATCGCCG
MYB31_UTR       AGGACGAGCTCATCGTCAAGCTGCACAGCGTCCTCGGCAACAAGTGGTCCCTGATCGCCG
NM_001112479    AGGACGAGCTCATCGTCAAGCTGCACAGCGTCCTCGGCAACAAGTGGTCCCTGATCGCCG
                ************************************************************

MYB31_ORF       GAAGGCTGCCCGGCAGGACGGACAACGAGATCAAGAACTACTGGAACACGCACATCCGGA
MYB31_UTR       GAAGGCTGCCCGGCAGGACGGACAACGAGATCAAGAACTACTGGAACACGCACATCCGGA
NM_001112479    GAAGGCTGCCCGGCAGGACGGACAACGAGATCAAGAACTACTGGAACACGCACATCCGGA
                ************************************************************
MYB31_ORF       GGAAGCTGCTGAGCAGGGGGATCGACCCGGTGACGCACCGCCCGGTCACGGAGCACCACG
MYB31_UTR       GGAAGCTGCTGAGCAGGGGGATCGACCCGGTGACGCACCGCCCGGTCACGGAGCACCACG
NM_001112479    GGAAGCTGCTGAGCAGGGGGATCGACCCGGTGACGCACCGCCCGGTCACGGAGCACCACG
                ************************************************************

MYB31_ORF       CGTCCAACATCACCATATCGTTCGAGACGGAGGTGGCCGCCGCCGCCCGTGATGATAAGA
MYB31_UTR       CGTCCAACATCACCATATCGTTCGAGACGGAGGTGGCCGCCGCCGCCCGTGATGATAAGA
NM_001112479    CGTCCAACATCACCATATCGTTCGAGACGGAAGTGGCCGCCGCTGCCCGTGATGATAAGA
                ******************************* *********** ****************

MYB31_ORF       AGGGCGCCGTCTTCCGGCTGGAGGACGAGGAG---------CGCAACAAGGCGACGATGG
MYB31_UTR       AGGGCGCCGTCTTCCGGCTGGAGGACGAGGAG---------CGCAACAAGGCGACGATGG
NM_001112479    AGGGCGCCGTCTTCCGGTTGGAGGACGAGGAGGAGGAGGAGCGCAACAAGGCGACGATGG
                ***************** **************         *******************

MYB31_ORF       TCGTCGGCCGCGACCGGCAGAGCCAGAGCCACAGCCACAGCCACCCCGCCGGCGAGTGGG
MYB31_UTR       TCGTCGGCCGCGACCGGCAGAGCCAGAGCCACAGCCACAGCCACCCCGCCGGCGAGTGGG
NM_001112479    TCGTCGGCCGCGACCGGCAGAGCCAGAGCCACAGCCACAGCCACCCCGCCGGCGAGTGGG
                ************************************************************

MYB31_ORF       GCCAGGGGAAGAGGCCGCTCAAGTGCCCCGACCTCAACCTGGACCTCTGCATCAGCCCGC
MYB31_UTR       GCCAGGGGAAGAGGCCGCTCAAGTGCCCCGACCTCAACCTGGACCTCTGCATCAGCCCGC
NM_001112479    GCCAGGGGAAGAGGCCGCTCAAGTGCCCCGACCTCAACCTGGACCTCTGCATCAGCCCGC
                ************************************************************
```

```
MYB31_ORF       CGTGCCAGGAGGAGGAGGAGATGGAGGAGGCTGCGATGAGAGTGAGACCGGCGGTGAAGC
MYB31_UTR       CGTGCCAGGAGGAGGAGGAGATGGAGGAGGCTGCGATGAGAGTGAGACCGGCGGTGAAGC
NM_001112479    CGTGCCAGGAGGAGGAGGAGATGGAGGAGGCTGCGATGAGAGTGAGACCGGCGGTGAAGC
                ************************************************************

MYB31_ORF       GGGAGGCCGGGCTCTGCTTCGGCTGCAGCCTGGGGCTCCCCAGGACCGCGGACTGCAAGT
MYB31_UTR       GGGAGGCCGGGCTCTGCTTCGGCTGCAGCCTGGGGCTCCCCAGGACCGCGGACTGCAAGT
NM_001112479    GGGAGGCCGGGCTCTGCTTCGGCTGCAGCCTGGGGCTCCCCAGGACCGCGGACTGCAAGT
                ************************************************************

MYB31_ORF       GCAGCAGCAGCAGCTTCCTCGGGCTCAGGACCGCCATGCTCGACTTCAGAAGCCTCGAGA
MYB31_UTR       GCAGCAGCAGCAGCTTCCTCGGGCTCAGGACCGCCATGCTCGACTTCAGAAGCCTCGAGA
NM_001112479    GCAGCAGCAGCAGCTTCCTCGGGCTCAGGACCGCCATGCTCGACTTCAGAAGCCTCGAGA
                ************************************************************

MYB31_ORF       TGAAATGA----------------------------------------------------
MYB31_UTR       TGAAATGAGCGCGCTTCTACCCTCTCTGTGTAGCTTCTCCCCCCCGTCGTCCTCGTTTTT
NM_001112479    TGAAATGAGCGCGCTTCT-CCCTCTCTGTGTAGCTTCTCCCCCCCGTCGTCCTCGTTTTT
                ****************** *****************************************

MYB31_ORF       ------------------------------------------------------------
MYB31_UTR       GTTTTGCCACACCTCACATGGATGATGAATTGATGATACGTGGTTGGTTAGTTTTTTCGT
NM_001112479    GTTTTGCCACACCTCACATGGATGATGAATTGATGATACGTGGTTGGTTAGTTTTTTCGT
                ************************************************************

MYB31_ORF       ------------------------------------------------------------
MYB31_UTR       AGGTGAAAAATACGCGATGGTGAGCGAGTGAAAGAGAGATTTTGTGCCCTGGGTCCTCCT
NM_001112479    AGGTGAAAAATACGCGATGGTGAGCGCGTGAAAGAGAGATTTTG-GCCCTGGGTCCTCCT
                ************************** ***************** ***************

MYB31_ORF       ------------------------------------------------------------
MYB31_UTR       CCCTGCTCTCTCGTGGTGCCCCATTGCGCCTCCTCTGTCCCCCCTCTCTCTCTCTGTATC
NM_001112479    CCGTGCTCTCTCGTGGTGCCCCATTGCGCCTCCTCTGTCCCCCCTCTCTCTCTCTGTATC
                ** *********************************************************

MYB31_ORF       ------------------------------------------------------------
MYB31_UTR       TCTGTAATCACCATCGCCA-----------------------------------------
NM_001112479    TCTGTAATCACCATCGCCAAATGATCATGGGGGGCAATATAATATAATACATGCTGCTAA
                *******************

MYB31_ORF       ------
MYB31_UTR       ------
NM_001112479    TGCTAT
```

S1b: MYB31 amino acid sequence alignment

```
MYB31_ORF       MGRSPCCEKAHTNKGAWTKEEDERLVAHIRAHGEGCWRSLPKAAGLLRCGKSCRLRWINY
MYB31_UTR       MGRSPCCEKAHTNKGAWTKEEDERLVAHIRAHGEGCWRSLPKAAGLLRCGKSCRLRWINY
NM_001112479    MGRSPCCEKAHTNKGAWTKEEDERLVAHIRAHGEGCWRSLPKAAGLLRCGKSCRLRWINY
                ************************************************************

MYB31_ORF       LRPDLKRGNFTEEEDELIVKLHSVLGNKWSLIAGRLPGRTDNEIKNYWNTHIRRKLLSRG
MYB31_UTR       LRPDLKRGNFTEEEDELIVKLHSVLGNKWSLIAGRLPGRTDNEIKNYWNTHIRRKLLSRG
NM_001112479    LRPDLKRGNFTEEEDELIVKLHSVLGNKWSLIAGRLPGRTDNEIKNYWNTHIRRKLLSRG
                ************************************************************

MYB31_ORF       IDPVTHRPVTEHHASNITISFETEVAAAARDDKKGAVFRLEDEE---RNKATMVVGRDRQ
MYB31_UTR       IDPVTHRPVTEHHASNITISFETEVAAAARDDKKGAVFRLEDEE---RNKATMVVGRDRQ
NM_001112479    IDPVTHRPVTEHHASNITISFETEVAAAARDDKKGAVFRLEDEEEEERNKATMVVGRDRQ
                ********************************************   *************

MYB31_ORF       SQSHSHSHPAGEWGQGKRPLKCPDLNLDLCISPPCQEEEEMEEAAMRVRPAVKREAGLCF
MYB31_UTR       SQSHSHSHPAGEWGQGKRPLKCPDLNLDLCISPPCQEEEEMEEAAMRVRPAVKREAGLCF
NM_001112479    SQSHSHSHPAGEWGQGKRPLKCPDLNLDLCISPPCQEEEEMEEAAMRVRPAVKREAGLCF
                ************************************************************

MYB31_ORF       GCSLGLPRTADCKCSSSSFLGLRTAMLDFRSLEMK
MYB31_UTR       GCSLGLPRTADCKCSSSSFLGLRTAMLDFRSLEMK
NM_001112479    GCSLGLPRTADCKCSSSSFLGLRTAMLDFRSLEMK
                ***********************************
```

S1c: MYB42 nucleotide sequence alignment

```
MYB42_ORF       ------------------------------------------------------------
MYB42_UTR       --------------------------------------ACTCGCTGCCTTCTCAAATCCA
NM_001112539    CGGGCGCAGGAATTCGGCACGAGGGGAAACCCGCGCCCACTCGCTGCCTTCTCAAATCCA
                                                      **********************

MYB42_ORF       ------------------------------------------ATGGGGCGGTCGCCGTGC
MYB42_UTR       AACGCGAAGTAGCAACAAGCAAAAGCCCAGATCGATAATACGATGGGGCGGTCGCCGTGC
NM_001112539    AACGCGAAGTAGCAACAAGCAAAAGCCCAGATCGATAATACGATGGGGCGGTCGCCGTGC
                ************************************************************

MYB42_ORF       TGCGAGAAGGCGCACACCAACAGGGGCGCGTGGACCAAGGAGGAGGACGAGCGGCTGGTG
MYB42_UTR       TGCGAGAAGGCGCACACCAACAGGGGCGCGTGGACCAAGGAGGAGGACGAGCGGCTGGTG
NM_001112539    TGCGAGAAGGCGCACACCAACAGGGGCGCGTGGACCAAGGAGGAGGACGAGCGGCTGGTG
                ************************************************************

MYB42_ORF       GCCTACGTCCGCGCGCACGGCGAAGGGTGCTGGCGCTCGCTGCCCAGGGCGGCGGGCCTG
MYB42_UTR       GCCTACGTCCGCGCGCACGGCGAAGGGTGCTGGCGCTCGCTGCCCAGGGCGGCGGGCCTG
NM_001112539    GCCTACGTCCGCGCGCACGGCGAAGGGTGCTGGCGCTCGCTGCCCAGGGCGGCGGGCCTG
                ************************************************************

MYB42_ORF       CTGCGCTGCGGCAAGAGCTGCCGCCTGCGCTGGATCAACTACCTCCGCCCGGACCTCAAG
MYB42_UTR       CTGCGCTGCGGCAAGAGCTGCCGCCTGCGCTGGATCAACTACCTCCGCCCGGACCTCAAG
NM_001112539    CTGCGCTGCGGCAAGAGCTGCCGCCTGCGCTGGATCAACTACCTCCGCCCGGACCTCAAG
                ************************************************************

MYB42_ORF       CGAGGCAACTTCACCGCCGACGAGGACGACCTCATCGTCAAGCTGCACAGCCTGCTCGGG
MYB42_UTR       CGCGGCAACTTCACCGCCGACGAGGACGACCTCATCGTCAAGCTGCACAGCCTGCTCGGG
NM_001112539    CGAGGCAACTTCACCGCCGACGAGGACGACCTCATCGTCAAGCTGCACAGCCTCCTCGGG
                ** ************************************************** ******
MYB42_ORF       AACAAGTGGTCGCTCATCGCCGCGCGGCTCCCGGGGCGGACGGACAACGAGATCAAGAAC
MYB42_UTR       AACAAGTGGTCGCTCATCGCCGCGCGGCTCCCGGGGCGGACGGACAACGAGATCAAGAAC
NM_001112539    AACAAGTGGTCGCTCATCGCCGCGCGGCTCCCGGGGCGGACGGACAACGAGATCAAGAAC
                ************************************************************

MYB42_ORF       TACTGGAACACGCACATCCGGCGCAAGCTGCTGTGCAGCGGCATCGACCCCGTCACGCAC
MYB42_UTR       TACTGGAACACGCACATCCGGCGCAAGCTGCTGTGCAGCGGCATCGACCCCGTCACGCAC
NM_001112539    TACTGGAACACGCACATCCGGCGCAAGCTGCTGGGCAGCGGCATCGACCCCGTCACGCAC
                ********************************* **************************

MYB42_ORF       CGCCGTGTCGCGGGTGGCGCCGCGACCACCATCTCGTTCCAGCCCAGCCCCAACTCCGCC
MYB42_UTR       CGCCGTGTCGCGGGTGGCGCCGCGACCACCATCTCGTTCCAGCCCAGCCCCAACTCCGCC
NM_001112539    CGCCGCGTCGCGGGGGGCGCCGCGACCACCATCTCGTTCCAGCCCAGCCCCAACTCCGCC
                ***** ******** *********************************************

MYB42_ORF       GCCGCCGCCGCC---GCAGAAGCAGCAGCGCAGGCGCCGATCAAGGCCGAGGAGACGGCG
MYB42_UTR       GCCGCCGCCGCC---GCAGAAGCAGCAGCGCAGGCGCCGATCAAGGCCGAGGAGACGGCG
NM_001112539    GCCGCCGCCGCCGCCGCAGAAACAGCAGCGCAGGCGCCGATCAAGGCCGAGGAGACGGCG
                ************   ****** **************************************

MYB42_ORF       GGCGTCAAGGCGCCCAGGTGCCCTGACCTCAACCTGGACCTCTGCATCAGCCCGCCGTGC
MYB42_UTR       GGCGTCAAGGCGCCCAGGTGCCCTGACCTCAACCTGGACCTCTGCATCAGCCCGCCGTGC
NM_001112539    GCCGTCAAGGCGCCCAGGTGCCCCGACCTCAACCTGGACCTCTGCATCAGCCCGCCGTGC
                * ********************* ************************************

MYB42_ORF       CAGCATGAGGACGACGGCGAGGAGGAGGACGAGGAGCTGGACCTCAAGCCCGCCTTCGTC
MYB42_UTR       CAGCATGAGGACGACGGCGAGGAGGAGGACGAGGAGCTGGACCTCAAGCCCGCCTTCGTC
NM_001112539    CAGCATGAGGACGACGGCGAGGAGGAGGACGAGGAGCTGGACCTCAAGCCCGCCTTCGTC
                ************************************************************

MYB42_ORF       AAGCGGGAGGCGCTGCAGGCCGGCCACGGCCACGGCCACGGCCTCTGCCTCGGCTGCGGC
MYB42_UTR       AAGCGGGAGGCGCTGCAGGCCGGCCACGGCCACGGCCACGGCCTCTGCCTCGGCTGCGGC
NM_001112539    AAGCGGGAGGCGCTGCAGGCCGGCCACGGCCACGGCCACGGCCTCTGCCTCGGCTGCGGC
                ************************************************************
```

```
MYB42_ORF       CTGGGCGGACAGAAGGGAGCGGCCGGGTGCAGCTGCAGCAACGGCCACCACTTCCTGGGG
MYB42_UTR       CTGGGCGGACAGAAGGGAGCGGCCGGGTGCAGCTGCAGCAACGGCCACCACTTCCTGGGG
NM_001112539    CTGGGCGGACAGAAGGGAGCGGCCGGGTGCAGCTGCAGCAACGGCCACCACTTCCTGGGG
                ************************************************************

MYB42_ORF       CTCAGGACCAGCGTGCTCGACTTCAGAGGCCTGGAGATGAAGTGA---------------
MYB42_UTR       CTCAGGACCAGCGTGCTCGACTTCAGAGGCCTGGAGATGAAGTGAACGAAACGAAGCCCA
NM_001112539    CTCAGGACCAGCGTGCTCGACTTCAGAGGCCTGGAGATGAAGTGAACGAAACGAAGCCCA
                ************************************************************

MYB42_ORF       ------------------------------------------------------------
MYB42_UTR       CACGTCCTTTCTTCTCC-------------------------------------------
NM_001112539    CACGTCCTTTCTTCTCCTTTTGTTGTCGGTTGTAGTCTTGGCTTGTTGGATTTGGATAGA
                *****************

MYB42_ORF       ------------------------------------------------------------
MYB42_UTR       ------------------------------------------------------------
NM_001112539    GCTAGTTGGTTACTAGTTGTTAGTTAGAAGATAGTGCAGGATGATCACTAGCTACTGGCT

MYB42_ORF       ------------------------------------------------------------
MYB42_UTR       ------------------------------------------------------------
NM_001112539    ACCTCAACAGTACAGTAGCTGCTCCCTTCTCTTCCATTCTATGTAAAAAAGAAACAAAAA

MYB42_ORF       -------------------------------------------------
MYB42_UTR       -------------------------------------------------
NM_001112539    TACTTATAAGGTGTTTGGGTTGAGAAATGAACTAGTCTATTATCTTTTC
```

S1d: MYB42 amino acid sequence alignment

```
MYB42_ORF       MGRSPCCEKAHTNRGAWTKEEDERLVAYVRAHGEGCWRSLPRAAGLLRCGKSCRLRWINY
MYB42_UTR       MGRSPCCEKAHTNRGAWTKEEDERLVAYVRAHGEGCWRSLPRAAGLLRCGKSCRLRWINY
NM_001112539    MGRSPCCEKAHTNRGAWTKEEDERLVAYVRAHGEGCWRSLPRAAGLLRCGKSCRLRWINY
                ************************************************************

MYB42_ORF       LRPDLKRGNFTADEDDLIVKLHSLLGNKWSLIAARLPGRTDNEIKNYWNTHIRRKLLCSG
MYB42_UTR       LRPDLKRGNFTADEDDLIVKLHSLLGNKWSLIAARLPGRTDNEIKNYWNTHIRRKLLCSG
NM_001112539    LRPDLKRGNFTADEDDLIVKLHSLLGNKWSLIAARLPGRTDNEIKNYWNTHIRRKLLGSG
                ********************************************************* **

MYB42_ORF       IDPVTHRRVAGGAATTISFQPSPNSAAAAA-AEAAAQAPIKAEETAGVKAPRCPDLNLDL
MYB42_UTR       IDPVTHRRVAGGAATTISFQPSPNSAAAAA-AEAAAQAPIKAEETAGVKAPRCPDLNLDL
NM_001112539    IDPVTHRRVAGGAATTISFQPSPNSAAAAAAAETAAQAPIKAEETAAVKAPRCPDLNLDL
                ****************************** **:************.*************

MYB42_ORF       CISPPCQHEDDGEEEDEELDLKPAFVKREALQAGHGHGHGLCLGCGLGGQKGAAGCSCSN
MYB42_UTR       CISPPCQHEDDGEEEDEELDLKPAFVKREALQAGHGHGHGLCLGCGLGGQKGAAGCSCSN
NM_001112539    CISPPCQHEDDGEEEDEELDLKPAFVKREALQAGHGHGHGLCLGCGLGGQKGAAGCSCSN
                ************************************************************

MYB42_ORF       GHHFLGLRTSVLDFRGLEMK
MYB42_UTR       GHHFLGLRTSVLDFRGLEMK
NM_001112539    GHHFLGLRTSVLDFRGLEMK
                ********************
```
