# Supplementary material for: Sugarcane transgenics expressing MYB transcription factors show improved glucose release
Source: Biotechnol Biofuels. 2016 Jul 15;9:143. doi: 10.1186/s13068-016-0559-1 (PMC4946106; doi:10.1186/s13068-016-0559-1)
Supplement: Supplementary file 2 — 10.1186/s13068-016-0559-1 Normalized MYB genes ΔCt values. Mean ΔCt value for MYB31 and MYB42 genes. Values represent post-harvest expression results from young internode tissue and maturing internode tissue. SE: standard error of the mean. Control n = 3. Letter groupings were determined by ANOVA followed by LSD test. [file 13068_2016_559_MOESM2_ESM.pdf]

Table S1 Normalised MYB genes ΔCt values.

		Young		Maturing	
		**Mean**	**SE**	**Mean**	**SE**
Control		0	0	0	0
MYB31 ORF	13	1.34[a]	0.14	2.19[c]	0.04
	11	1.10[ab]	0.21	1.49[c]	0.02
	2	0.65[c]	0.02	0.30[d]	0.01
	7	0.50[c]	0.01	2.55[bc]	0.24
	1	0.06[d]	0.00	0.29[d]	0.01
	8	0[d]	0	4.87[ab]	1.51
	9	0.78[bc]	0.10	5.42[a]	1.12
MYB31 UTR	27	1.56[b]	0.04	1.53[d]	0.09
	2	1.25[b]	0.03	3.73[b]	0.05
	18	1.58[b]	0.18	2.83[c]	0.07
	11	0.02[d]	0.00	0.16[f]	0.00
	12	11.00[a]	1.16	5.53[a]	0.70
	7	0.10[c]	0.01	0.45[e]	0.03
	20	0.09[c]	0.00	0.32e[f]	0.00
MYB42 ORF	14	0.81[a]	0.03	4.85[a]	0.12
	16	0.56[c]	0.02	1.60[b]	0.09
	23	0.67[b]	0.02	1.23[c]	0.11
	11	0.06[f]	0.00	0.34[d]	0.00
	18	0.29[d]	0.03	1.12[c]	0.02
	21	0.12[e]	0.01	0.28[e]	0.00
	26	0.03[f]	0.00	0.17[f]	0.01
MYB42 UTR	28	0.26[bc]	0.03	0.36[c]	0.00
	6	0.45[a]	0.04	1.29[a]	0.04
	32	0.32[b]	0.01	0.23[d]	0.01
	30	0.24[c]	0.00	1.10[b]	0.06
	15	0.12[d]	0.01	0.20[e]	0.01
	26	0.05[de]	0.00	0.15[f]	0.00
	16	0[e]	0	0.05[g]	0.00
