# Supplementary material for: Sugarcane transgenics expressing MYB transcription factors show improved glucose release
Source: Biotechnol Biofuels. 2016 Jul 15;9:143. doi: 10.1186/s13068-016-0559-1 (PMC4946106; doi:10.1186/s13068-016-0559-1)
Supplement: Supplementary file 4 — 10.1186/s13068-016-0559-1 MYB-expressing sugarcane cell wall composition. The percentage of each component of the total composition is shown with the standard error of the mean. Samples significantly different to the controls after ANOVA followed by LSD test, p = 0.05, are shown in bold. Control n = 3. Plants are listed in ascending total lignin content for each line. [file 13068_2016_559_MOESM4_ESM.pdf]

Table S3 MYB-expressing sugarcane cell wall composition.

		Total lignin		Acid-insoluble lignin		Acid-soluble lignin		Ash Content		Glucose		Xylose		Galactose		Arabinose	
		%	+/-	%	+/-	%	+/-	%	+/-	%	+/-	%	+/-	%	+/-	%	+/-
Control		23.37	0.39	17.92	0.38	5.45	0.10	0.13	0.07	47.17	0.45	20.23	0.28	0.10	0.08	1.69	0.22
MYB31 ORF	13	21.74	0.15	16.49	0.09	5.25	**0.18**	0.10	0.05	48.77	0.33	20.27	0.17	0.00	0.00	1.49	0.06
	11	22.23	0.15	16.60	0.17	5.64	0.11	0.19	0.02	45.45	0.41	22.05	0.15	1.24	0.02	2.95	0.03
	2	22.50	0.04	17.61	0.09	**4.89**	**0.07**	0.11	0.01	47.79	0.30	20.83	0.13	**1.05**	**0.02**	2.21	0.04
	7	23.07	0.30	17.61	0.22	5.45	0.12	0.18	0.02	46.74	0.61	**22.11**	**0.17**	0.00	0.00	2.24	0.06
	1	23.08	0.29	17.86	0.24	5.22	0.14	0.11	0.03	45.08	0.18	**23.14**	**0.18**	**0.98**	**0.01**	2.24	0.03
	8	23.88	0.40	18.47	0.40	5.41	0.04	0.04	0.08	46.01	0.82	21.34	0.37	**1.24**	**0.05**	2.71	0.07
	9	24.97	0.47	19.72	0.40	5.25	0.11	0.12	0.09	46.88	0.56	20.88	0.39	**1.07**	**0.05**	2.28	0.03
MYB31 UTR	27	**21.03**	**0.19**	16.29	0.14	**4.74**	**0.05**	0.27	0.05	**51.76**	**0.51**	**24.27**	**0.23**	0.00	0.00	2.19	0.05
	2	21.18	0.18	**15.63**	**0.12**	5.55	0.12	0.05	0.04	45.63	0.41	**22.03**	**0.32**	**1.33**	**0.02**	**3.02**	**0.05**
	18	21.48	0.19	**15.57**	**0.18**	5.91	0.08	0.08	0.04	47.40	0.36	**22.11**	**0.26**	**1.28**	**0.00**	2.71	0.06
	11	22.25	0.07	17.10	0.09	5.16	0.14	0.13	0.02	46.05	0.14	**22.44**	**0.08**	**1.06**	**0.05**	2.58	0.03
	12	22.40	0.16	17.01	0.04	5.39	0.15	0.06	0.05	47.12	0.31	20.56	0.14	0.00	0.00	2.06	0.02
	7	22.79	0.11	17.13	0.06	5.66	0.15	0.13	0.04	45.38	0.17	20.15	0.15	0.32	0.26	1.92	0.05
	20	22.86	0.07	17.47	0.05	5.40	0.12	0.13	0.02	47.60	0.15	21.38	0.13	0.00	0.00	1.84	0.08
MYB42 ORF	14	**18.51**	**0.13**	**12.84**	**0.07**	5.67	0.20	**0.36**	**0.03**	49.24	0.14	21.28	0.22	**1.08**	**0.02**	2.63	0.04
	16	**20.86**	**0.05**	**15.25**	**0.01**	5.61	0.04	0.16	0.06	**49.62**	**0.38**	20.81	0.08	0.32	0.26	2.14	0.03
	23	**21.58**	**0.22**	**15.85**	**0.25**	5.73	0.06	0.03	0.05	47.86	0.18	19.60	0.34	0.30	0.24	2.17	0.05
	11	22.05	0.09	16.94	0.12	5.11	0.07	0.06	0.05	47.92	0.38	**22.67**	**0.24**	**0.98**	**0.02**	2.08	0.02
	18	22.38	0.10	16.76	0.09	5.62	0.01	0.10	0.04	48.69	0.55	19.90	0.09	0.00	0.00	1.96	0.02
	21	22.47	0.14	17.74	0.07	**4.73**	**0.15**	0.19	0.01	48.85	1.12	21.82	0.38	0.00	0.00	1.21	0.08
	26	22.99	0.09	17.68	0.01	5.30	0.10	**0.29**	**0.06**	46.61	0.33	21.61	0.22	**1.10**	**0.06**	2.50	0.06
MYB42 UTR	28	**19.42**	**0.29**	**13.78**	**0.34**	5.64	0.05	0.05	0.06	**50.55**	**0.13**	21.78	0.48	0.63	0.26	2.60	0.04
	6	**20.21**	**0.03**	**14.40**	**0.06**	5.81	0.08	0.07	0.02	46.41	0.16	**23.28**	**0.14**	**1.15**	**0.04**	**3.03**	**0.01**
	32	**20.91**	**0.24**	**15.46**	**0.14**	5.45	0.14	0.23	0.06	47.83	0.29	21.53	0.11	0.28	0.23	1.96	0.05
	30	21.51	0.30	**15.29**	**0.19**	6.21	0.20	0.14	0.06	49.12	0.28	**23.94**	**0.23**	**1.21**	**0.02**	2.63	0.03
	15	22.57	0.10	16.73	0.08	5.84	0.06	0.07	0.01	47.10	0.31	21.59	0.22	0.00	0.00	1.72	0.07
	26	23.48	0.11	18.36	0.07	5.12	0.08	0.10	0.02	47.41	0.45	**23.58**	**0.28**	0.00	0.00	1.67	0.03
	16	24.15	0.21	18.98	0.14	5.17	0.08	0.21	0.03	47.06	0.31	20.89	0.18	0.00	0.00	1.41	0.04
