# Supplementary material for: Sugarcane transgenics expressing MYB transcription factors show improved glucose release
Source: Biotechnol Biofuels. 2016 Jul 15;9:143. doi: 10.1186/s13068-016-0559-1 (PMC4946106; doi:10.1186/s13068-016-0559-1)
Supplement: Supplementary file 5 — 10.1186/s13068-016-0559-1 Glucose release by enzymatic hydrolysis. Glucose released into enzymatic hydrolysis solution (mg/mL) per gram (g) of bagasse measured at six time points over 72 h. The glucose released is shown with standard error of the mean. Samples significantly different to the control group after ANOVA followed by LSD test, p = 0.05, are shown in bold. Control n = 3. [file 13068_2016_559_MOESM5_ESM.pdf]

Table S4 Glucose release by enzymatic hydrolysis.

		0 h		6 h		12 h		24 h		48 h		72 h	
		mg/mL	+/-	mg/mL	+/-	mg/mL	+/-	mg/mL	+/-	mg/mL	+/-	mg/mL	+/-
Control		1.02	0.09	24.61	1.31	29.79	0.69	36.18	1.15	40.65	0.85	40.97	0.83
MYB31 ORF	13	1.07	0.07	25.72	0.20	31.38	0.12	34.94	0.65	38.06	0.72	38.47	0.93
	11	1.09	0.00	26.39	0.22	31.51	0.21	37.63	1.04	36.99	0.31	39.76	0.29
	2	0.97	0.07	23.85	0.43	27.83	0.95	**30.80**	**0.70**	37.91	1.77	37.56	0.45
MYB31 UTR	27	0.87	0.07	23.03	0.13	28.44	0.70	32.83	0.38	**35.38**	**0.67**	37.71	0.42
	2	1.07	0.11	29.84	0.26	**38.52**	**0.62**	**43.01**	**0.85**	**48.86**	**0.82**	**50.96**	**0.13**
	18	1.22	0.08	29.23	0.10	**35.86**	**0.42**	**42.57**	**0.16**	**46.90**	**0.77**	**49.48**	**0.69**
MYB42 ORF	14	**1.57**	**0.06**	**34.29**	**0.15**	**41.06**	**0.41**	**47.41**	**0.35**	**53.89**	**1.49**	**58.43**	**1.71**
	16	1.38	0.02	**34.80**	**0.10**	**43.31**	**0.36**	**47.79**	**0.22**	**51.92**	**0.89**	**53.58**	**0.46**
	23	1.42	0.02	**33.18**	**0.69**	**43.03**	**1.23**	**44.61**	**0.59**	52.14	3.23	**51.76**	**0.70**
MYB42 UTR	28	0.77	0.05	31.44	0.19	**39.43**	**0.62**	**44.71**	**0.51**	**48.65**	**0.28**	**51.49**	**1.00**
	6	1.15	0.02	**33.01**	**0.55**	**42.89**	**0.45**	**49.95**	**1.70**	**54.11**	**0.90**	**57.53**	**1.70**
	32	0.79	0.09	27.49	0.09	**36.54**	**0.13**	42.03	0.28	**48.03**	**0.34**	**52.51**	**0.33**
