# Supplementary material for: Sugarcane transgenics expressing MYB transcription factors show improved glucose release
Source: Biotechnol Biofuels. 2016 Jul 15;9:143. doi: 10.1186/s13068-016-0559-1 (PMC4946106; doi:10.1186/s13068-016-0559-1)
Supplement: Supplementary file 6 — 10.1186/s13068-016-0559-1 Cellulose crystallinity index of MYB bagasse. CI was calculated from the height ratio between the intensity of the crystalline peak (I002–IAM) and total intensity (I002) after subtraction of the background signal. [file 13068_2016_559_MOESM6_ESM.pdf]

Table S5 Cellulose crystallinity index of MYB bagasse.

Plant		Crystallinity Index (%)
Control	8	49.03
Control	10	51.49
MYB31 ORF	27	45.56
MYB31 ORF	11	47.50
MYB31 UTR	18	45.60
MYB42 ORF	23	47.41
MYB42 UTR	32	45.97
