# Supplementary material for: Sugarcane transgenics expressing MYB transcription factors show improved glucose release
Source: Biotechnol Biofuels. 2016 Jul 15;9:143. doi: 10.1186/s13068-016-0559-1 (PMC4946106; doi:10.1186/s13068-016-0559-1)
Supplement: Supplementary file 7 — 10.1186/s13068-016-0559-1 Primers for ZmMYB cloning, genomic PCR and qPCR. Primers designed from GenBank accessions for PCR amplification of ZmMYB31 and ZmMYB42 genes from maize cDNA (1–4), for leaf gDNA screening from ZmMYB31 and ZmMYB42 gene cassettes (5–8) and qPCR quantification of MYB gene expression in young and maturing internodes (9–10). [file 13068_2016_559_MOESM7_ESM.pdf]

Table S6 Primers for *Zm*MYB cloning, genomic PCR and qPCR

	Amplicon	Accession	Forward (5'-3')	Reverse (5'-3')	Size (bp)
1	MYB31ORF	NM_001112479	ATGGGGAGGTCGCCGTGCTG	TCATTTCATCTCGAGGCTTCT	819
2	MYB31UTR	NM_001112479	ACAGCAGCAGCAACAACAAC	TGGCGATGGTGATTACAGAG	1198
3	MYB42ORF	NM_001112539	ATGGGGCGGTCGCCGTGCTGC	TCACTTCATCTCCAGGCCTCT	780
4	MYB42UTR	NM_001112539	ACTCGCTGCCTTCTCAAATC	GGAGAAGAAAGGACGTGTGG	876
5	MYB31ORF		GCGGTCGTTCATTCGTTCTA	GATCAGGGACCACTTGTTGC	699
6	MYB31UTR		GCGGTCGTTCATTCGTTCTA	GATCAGGGACCACTTGTTGC	827
7	MYB42ORF		GCGGTCGTTCATTCGTTCTA	GATGAGCGACCACTTGTTCC	699
8	MYB42UTR		GCGGTCGTTCATTCGTTCTA	GATGAGCGACCACTTGTTCC	763
9	MYB31		TCTTCCGGCTGGAGGACGAG	GTGGCTGTGGCTCTGGCTCTG	80
10	MYB42		ATCAAGGCCGAGGAGACGG	AGAGGTCCAGGTTGAGGTCAG	64
