# Supplementary material for: Sugarcane transgenics expressing MYB transcription factors show improved glucose release
Source: Biotechnol Biofuels. 2016 Jul 15;9:143. doi: 10.1186/s13068-016-0559-1 (PMC4946106; doi:10.1186/s13068-016-0559-1)
Supplement: Supplementary file 8 — 10.1186/s13068-016-0559-1 Primers for qPCR of lignin biosynthetic genes. [file 13068_2016_559_MOESM8_ESM.pdf]

Table S7 Primers for qPCR of lignin biosynthetic genes

Gene	Forward (5'-3')	Reverse (5'-3')	Size (bp)
β-Tubulin[1]	GGAGGAGTACCCTGACAGAATGA	CAGTATCGGAAACCTTTGGTGAT	68
PAL	GACATCCTGAAGCTCATGTCG	ACCGACGTCTTGATGTTCTCC	92
C4H	GTTCACCGTGTACGGCGACCACT	GAAGAAGGGCACCGTCATGATCC	61
4CL	CTTCCCGACATCGAGATCAACAAC	CTCATCTTCCCGAAGCAGTAGGC	62
C3H	GTCGACGAGCAGGTCTTCAAAGC	CGTGCTCCTCCATGATCTTCAC	73
CCoAOMT	ACCTCATCGCAGACGAGAAGAAC	AGCCGCTCGTGGTAGTTGAGGTAG	91
CCR	AGCAGCCGTACAAGTTCTCG	GAAGGTTCTTCACCGTGTCG	96
F5H	GGTTCATCGACAAGATCATCGAC	GTCGGGGCTCTTCCCGCGCTTCAC	53
COMT	TACGGGATGACGGCGTTCGAGTAC	GTGATGATGACCGAGTGGTTCTT	92
CAD	ATCAGCTCGTCGTCCAAGAAG	ACCGTGTCGATGATGTAGTCC	128
